# Nanobody‐Mediated c‐MYC Degradation Inhibits Tumor Cell Progression

**DOI:** 10.1002/mco2.70701

**Published:** 2026-03-26

**Authors:** Yuanyuan Xue, Hao Jiang, Zhaoyun Zong, Xiaolin Tian, Zelong Miao, Ting Li, Yali Wei, Haiteng Deng

**Affiliations:** ^1^ MOE Key Laboratory of Bioinformatics Center For Synthetic and Systematic Biology School of Life Sciences Tsinghua University Beijing China; ^2^ Department of Clinical Laboratory Shandong Provincial Hospital Jinan China; ^3^ Zhejiang Key Laboratory of Multiomics and Molecular Enzymology Yangtze Delta Region Institute of Tsinghua University Jiaxing China

**Keywords:** c‐MYC degradation, cell‐penetrating peptide (CPP), nanobody, targeted cancer therapy

## Abstract

The c‐MYC oncogene, a critical driver of malignancies, is frequently associated with poor prognosis because it promotes unchecked cell proliferation and alters gene expression. Effective targeting of c‐MYC using conventional therapeutic strategies has been difficult, largely because of its unstructured nature. In the present study, we identified a myc‐binding nanobody named as M4 from a synthetic phage‐display nanobody library. We conjugated M4 with a cell‐penetrating peptide (CPP) to generate a molecule CPM4 and examined the effects and action mechanisms of CPM4 in inhibition of tumor cell growth in vitro and in vivo. CPM4 exhibited efficient nuclear localization, caused c‐MYC reduction, and induced apoptosis in MYC‐expressing cells. Hydrogen/deuterium exchange mass spectrometry revealed that CPM4 binds to the central PEST sequence (241–263 epitope) of c‐MYC with high affinity. Further analysis revealed that CPM4 promotes c‐MYC degradation via enhanced phosphorylation at Thr58, disrupts the c‐MYC/MAX heterodimer, and downregulates c‐MYC‐targeted downstream genes. Xenograft studies further validated the therapeutic efficacy of CPM4, showing a significant reduction in tumor growth. These results underscore the therapeutic potential of CPM4 as an effective drug candidate for inhibiting c‐MYC‐driven tumor growth.

## Graphical abstract

A cell‐permeable nanobody strategy was developed to target the intrinsically disordered oncoprotein c‐MYC. A synthetic nanobody, M4, binds the central PEST region of c‐MYC (241–263) and promotes c‐MYC degradation by enhancing Thr58 phosphorylation. Conjugation of M4 to a cell‐penetrating peptide (CPP) generates CPM4, which efficiently accumulates in the nucleus, disrupts the c‐MYC/MAX heterodimer, downregulates MYC‐driven transcriptional programs, and induces apoptosis in MYC‐expressing tumor cells. Consequently, CPM4 suppresses tumor growth in vitro and in vivo, highlighting the therapeutic potential of nanobody‐based c‐MYC targeting.

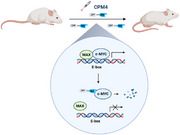

## 1 Introduction

Cancer remains a major cause of mortality worldwide and is driven by sustained proliferative signaling, reduced susceptibility to programmed cell death, and progressive acquisition of migratory/invasive traits [1, 2]. Among the molecular alterations that enable malignant transformation, deregulation of the c‐MYC oncogene is a prominent driver of tumorigenesis [3, 4, 5]. At the mechanistic level, c‐MYC functions as a transcriptional regulator that coordinates extensive gene‐expression networks governing cellular growth, metabolic state, and survival programs [6, 7, 8, 9]. In line with these broad functions, c‐MYC is frequently overexpressed or hyperactivated across diverse cancers, with representative examples including Burkitt's lymphoma, breast cancer, colorectal cancer, and hepatocellular carcinoma [10, 11, 12, 13, 14]. By coupling accelerated proliferation with metabolic rewiring, MYC confers strong oncogenic fitness, and elevated MYC activity has repeatedly been associated with aggressive disease features and unfavorable clinical outcomes [15, 16, 17, 18, 19].

Despite its therapeutic potential, c‐MYC's unconventional protein structure poses substantial challenges for traditional drug development [20, 21]. Unlike other oncogenic proteins, c‐MYC lacks well‐defined pockets or grooves for small‐molecule binding, rendering it “undruggable” by conventional methods [22, 23, 24]. Indirect strategies (e.g., upstream signaling blockade) can be bypassed by pathway rewiring, while direct dimerization/E‐box interference may incompletely extinguish MYC function and does not necessarily reduce MYC protein abundance [25, 26]. Together, these constraints motivate alternative modalities that engage MYC through orthogonal mechanisms with tunable selectivity and depth of target suppression.

Nanobodies, or single‐domain antibodies derived from camelid heavy‐chain antibodies, represent a next‐generation biologic format with distinct advantages [27, 28]. They retain the antigen‐binding specificity of conventional antibodies but are smaller and more stable, enhancing their ability to penetrate tissues and cellular compartments [29, 30, 31]. Their unique properties—high specificity, low immunogenicity, and the capacity to bind cryptic epitopes—imply that they could be ideal for targeting challenging proteins like c‐MYC [32, 33, 34, 35, 36]. A major barrier to fully exploiting nanobodies against nuclear targets is the need for efficient intracellular and intranuclear delivery while maintaining specificity and functional potency. CPPs have emerged as versatile carriers capable of transporting proteins, peptides, and nucleic acids across biological membranes [37, 38]. When covalently linked to nanobodies, CPPs can generate “cell‐permeable nanobodies” that traverse the plasma membrane, accumulate in the nucleus, and engage nuclear oncoproteins in their native context. Supporting this concept, we recently reported a cell‐permeable MYC nanobody (CPMycNB) generated by conjugating a MYC‐specific VHH to a CPP. CPMycNB enters tumor cell nuclei, blocks c‐MYC–MAX interaction, and suppresses MYC‐driven transcription. Thereby establishing a proof‐of‐principle for nanobody‐based targeting of c‐MYC in vivo [39]. While CPMycNB primarily acts by blocking c‐MYC–MAX dimerization and DNA binding, it does not consistently trigger robust c‐MYC protein degradation. It is important to use complementary nanobody designs targeting distinct functional domains and coupling MYC binding to degradation pathways to further expand the therapeutic potential of MYC‐directed biologics. In particular, strategies that promote phosphorylation‐dependent proteasomal turnover of c‐MYC could provide deeper and more durable suppression of MYC‐driven oncogenic programs [40, 41].

In this study, we established a synthetic phage‐display nanobody library and identified M4, a nanobody with high affinity for c‐MYC. We conjugated M4 with a CPP to generate a cell‐permeable molecule CPM4, which efficiently reduces c‐MYC protein abundance, suppresses tumor cell proliferation, and induces apoptosis. We provided deeper mechanistic insight, expanding validation to additional cell lines and functional readouts, optimizing a degradation‐oriented nanobody design, and establishing CPM4 as a modality for c‐MYC‐dependent tumor treatment.

## 2 Results

### 2.1 Identification c‐MYC‐Targeting Nanobody

**FIGURE 1:**
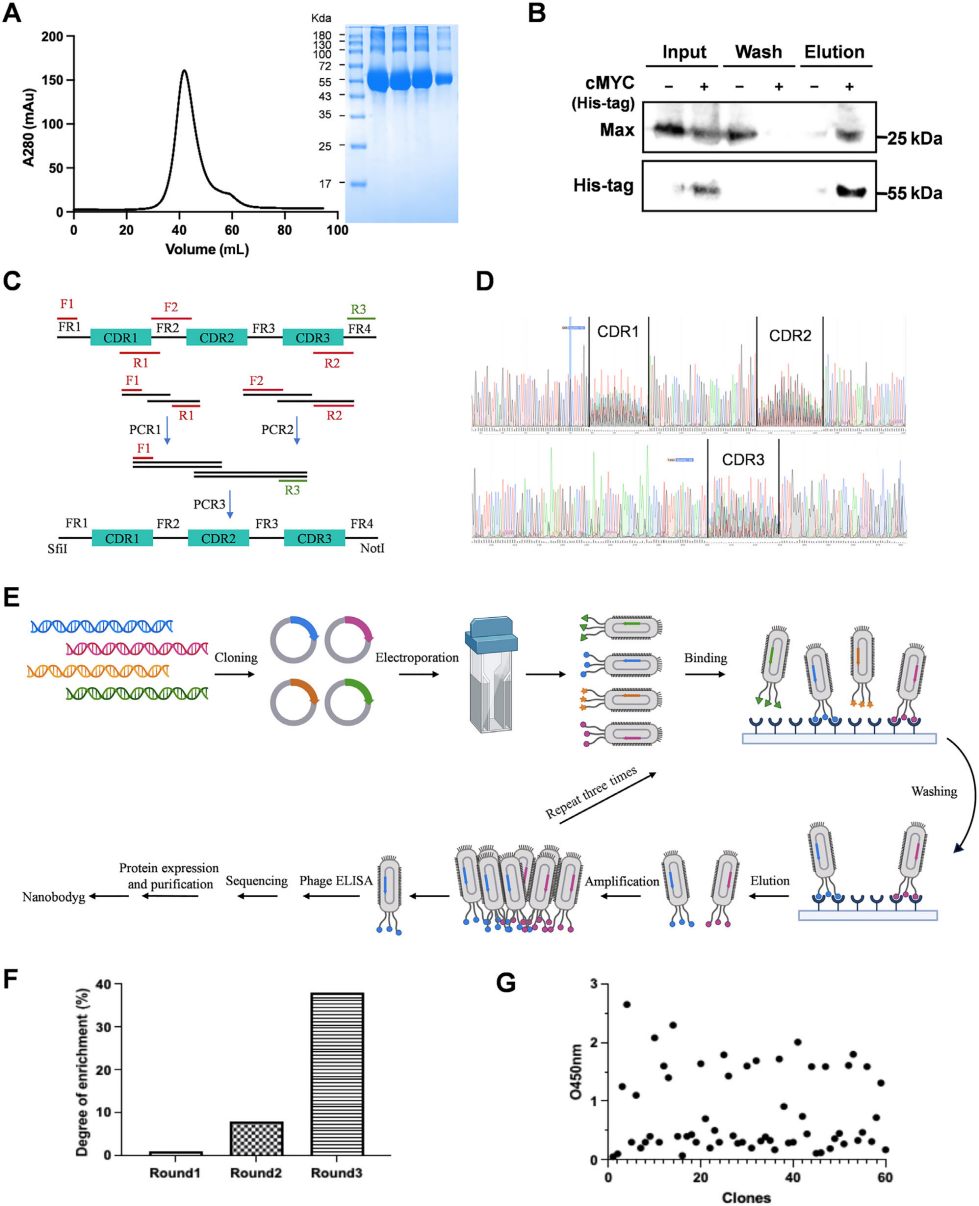
Generation of a synthetic nanobody library targeting c‐MYC. (A) Size‐exclusion chromatogram and SDS‐PAGE analysis of recombinant full‐length human c‐MYC purified from E. coli inclusion bodies. (B) Pull‐down of endogenous MAX from 293T cell lysates using His‐tagged c‐MYC immobilized on Ni‐NTA resin, analyzed by Western blot; beads without c‐MYC serve as a negative control. (C) Schematic representation of the design and overlap‐extension PCR strategy used to generate synthetic VHH genes with diversified CDRs. (D) Representative Sanger sequencing chromatograms illustrating sequence diversity within CDR1, CDR2, and CDR3 in the synthetic nanobody library. (E) Workflow for construction and phage display of the anti‐c‐MYC VHH library, including library cloning, phage rescue, and panning on immobilized c‐MYC. (F) Phage output titers over three successive rounds of biopanning on c‐MYC, showing progressive enrichment of c‐MYC‐binding clones. (G) Phage ELISA screening of individual clones after panning; 60 positive clones were identified based on OD450 values at least twofold higher than the negative control.

Although c‐MYC is widely considered an intrinsically disordered protein, it comprises discrete functional elements, including an N‐terminal regulatory segment and a C‐terminal basic helix–loop–helix leucine‐zipper region that acquires greater structural order upon heterodimerization with MAX. To maximize antigen specificity and preserve the full repertoire of potential c‐MYC epitopes, we generated a full‐length human c‐MYC expression construct for bacterial production. This construct was expressed in *E. coli* and purified from inclusion bodies, yielding c‐MYC with 90% purity (Figure 1A). We next evaluated whether the purified c‐MYC preserved its capacity to interact with endogenous partners. His‐tagged c‐MYC immobilized on Ni‐NTA resin was incubated with 293T cell lysates, and bound proteins were eluted after extensive washing. Western blotting revealed a strong MAX signal specifically in the c‐MYC‐containing fraction, whereas control beads displayed minimal background, confirming that the recombinant antigen adopts a conformation competent for MAX binding and is suitable for phage‐display selection (Figure 1B).

A fully synthetic nanobody library was designed and constructed using the framework region (FR) sequences derived from llamas, known for their favorable expression characteristics [42, 43, 44, 45]. To define the backbone and diversification positions, representative VHH sequences from natural repertoires were aligned, enabling annotation of FRs and complementarity‐determining regions (CDRs). These sequences were optimized for *E. coli* codon usage and were inserted into the plasmid pCantab5E. This plasmid served as the template for synthetic VHHs during three rounds of PCR, with NNK mutations introduced into the CDR regions to enhance diversity [46, 47] (Figure 1C). The calculations confirmed that a library containing 3 × 10^1^ ^1^ CFU had been successfully created. Quality control by colony PCR of 100 randomly picked clones verified the expected amplification of FR1–FR4 and the presence of appropriately sized CDR1–CDR3 segments (Figure 1D). These results indicate that the library exhibits substantial diversity, which is essential for effective phage display screening.

The phage display library was amplified using the M13KO7 helper phage and subjected to successive rounds of panning on a MaxiSorp plate with purified human c‐MYC immobilized in nonadjacent wells (Figure 1E). After three rounds, the enrichment of positive phage clones targeting c‐MYC‐specific VHH showed a 40‐fold increase compared with the initial round (Figure 1F). Phage enzyme‐linked immunosorbent assay (ELISA) identified 60 clones with OD450 values that were twice those of the blank control, confirming that there was specific binding to c‐MYC (Figure 1G).

### 2.2 Functionalization of the M4 Nanobody for Cell Penetration

**FIGURE 2:**
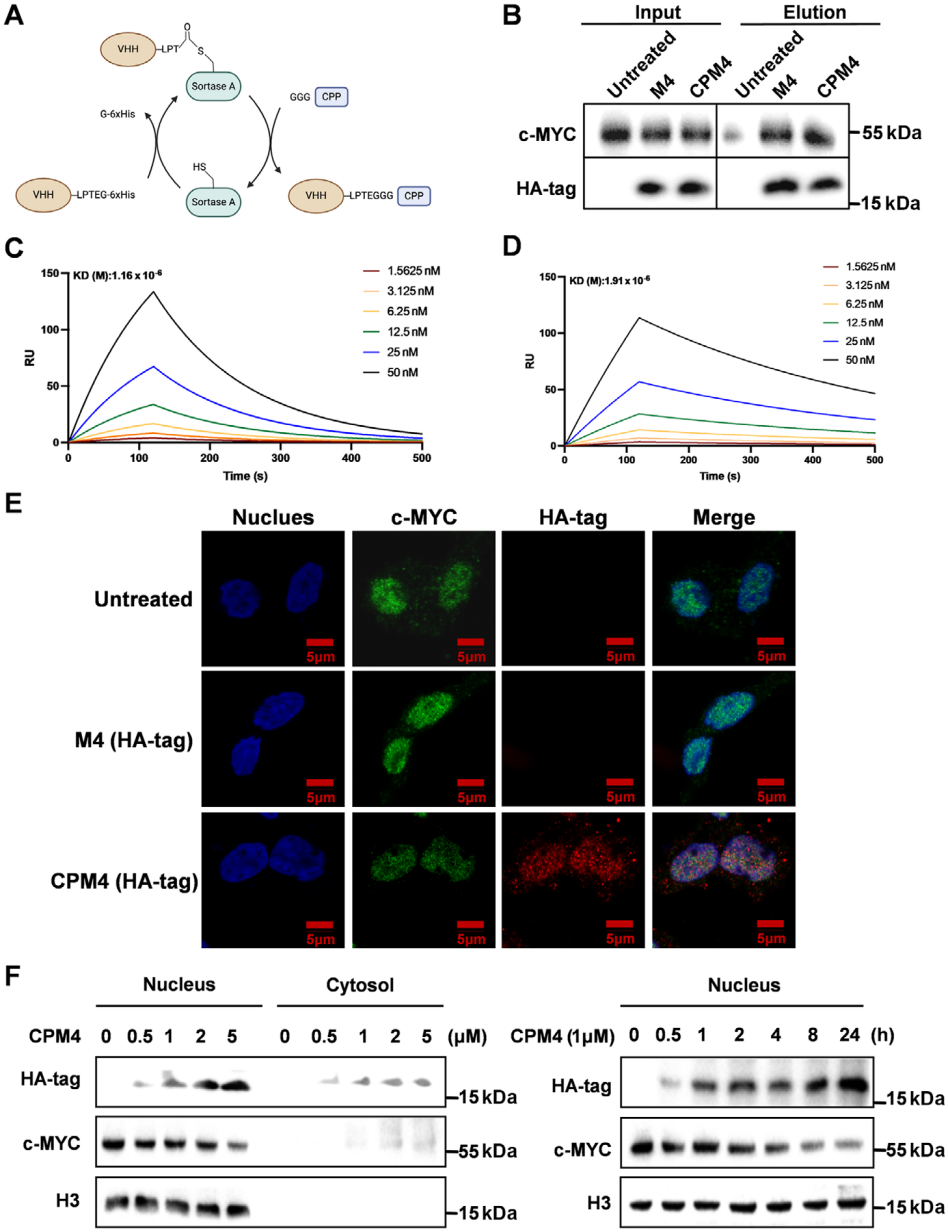
Biochemical and cellular characterization of the M4 nanobody and its cell‐permeable conjugate CPM4. (A) Schematic representation of the Sortase A‐mediated, site‐specific conjugation strategy. An LPETG‐tagged VHH (M4) is coupled to an N‐terminal GGG‐containing cell‐penetrating peptide (CPP) to generate the CPM4 conjugate. (B) Coimmunoprecipitation of endogenous c‐MYC from HCT116 cell lysates using M4 or CPM4, followed by immunoblotting, confirming specific binding to c‐MYC. (C and D) Surface plasmon resonance (SPR) sensorgrams showing the binding of M4 (C) and CPM4 (D) to immobilized c‐MYC at increasing analyte concentrations, demonstrating high‐affinity interactions that are preserved after CPP conjugation. (E) Confocal microscopy of HCT116 cells treated with CPM4. Nuclei are stained with DAPI (blue), c‐MYC is detected by immunofluorescence (green), CPM4 is visualized in red, and merged images show nuclear localization and colocalization of CPM4 with c‐MYC. Scale bars, 5 µm. (F) Immunoblot analysis of CPM4 uptake in HCT116 cells, showing concentration‐dependent and time‐dependent accumulation of CPM4 in cell lysates; histone H3 serves as a loading control.

Four candidate MYC nanobodies were selected, and their expression, purification, and binding affinities to c‐MYC were analyzed (Figure S1A–D,G). M4, the clone with the highest number of reads and the strongest binding affinity to c‐MYC (Figure 2B), was selected for further investigation. It exhibited good solubility and homogeneity on SDS‐PAGE, showing a single band at approximately 15 kDa (Figure S1A). The expression yield after IPTG induction was approximately 12.6 mg/L of bacterial culture.

Because c‐MYC resides primarily in the nucleus, direct intracellular delivery of M4 is essential for functional interference. We therefore adopted a transmembrane delivery strategy in which M4 carrying a C‐terminal LPETG motif and His tag (M4–LPETG–6×His) was produced in Escherichia coli and coupled to N‐terminal GGG‐containing CPPs using Sortase A‐mediated transpeptidation [48, 49] (Figure 2A). Both Sortase A enzyme and M4–LPETG–6xHis were successfully expressed in *E. coli* and purified, facilitating the coupling of M4 with CPPs (Figure S1E). MALDI–TOF mass spectrometry confirmed successful conjugation, with the peak shifting from 15.8 kDa before conjugation to 16.7 kDa after conjugation (**Figure** **S2C**). Importantly, pull‐down assays and surface plasmon resonance (SPR) analysis showed that CPP fusion did not measurably compromise M4 binding to c‐MYC (Figure 2B,D). Confocal microscopy further revealed that the CPM4 conjugate was successfully localized to the nuclei of HCT116 cells postincubation, indicating effective nuclear targeting (Figure 2E). Immunoblot analysis confirmed the dose‐dependent uptake of CPM4 by cells (Figure 2F). These results indicate that CPP conjugation does not disrupt the binding interface or recognition mechanisms between M4 and c‐MYC, thereby supporting its potential for efficient intracellular delivery and targeting.

### 2.3 Characterization of c‐MYC Antigenic Epitopes Recognized by CPM4

**FIGURE 3:**
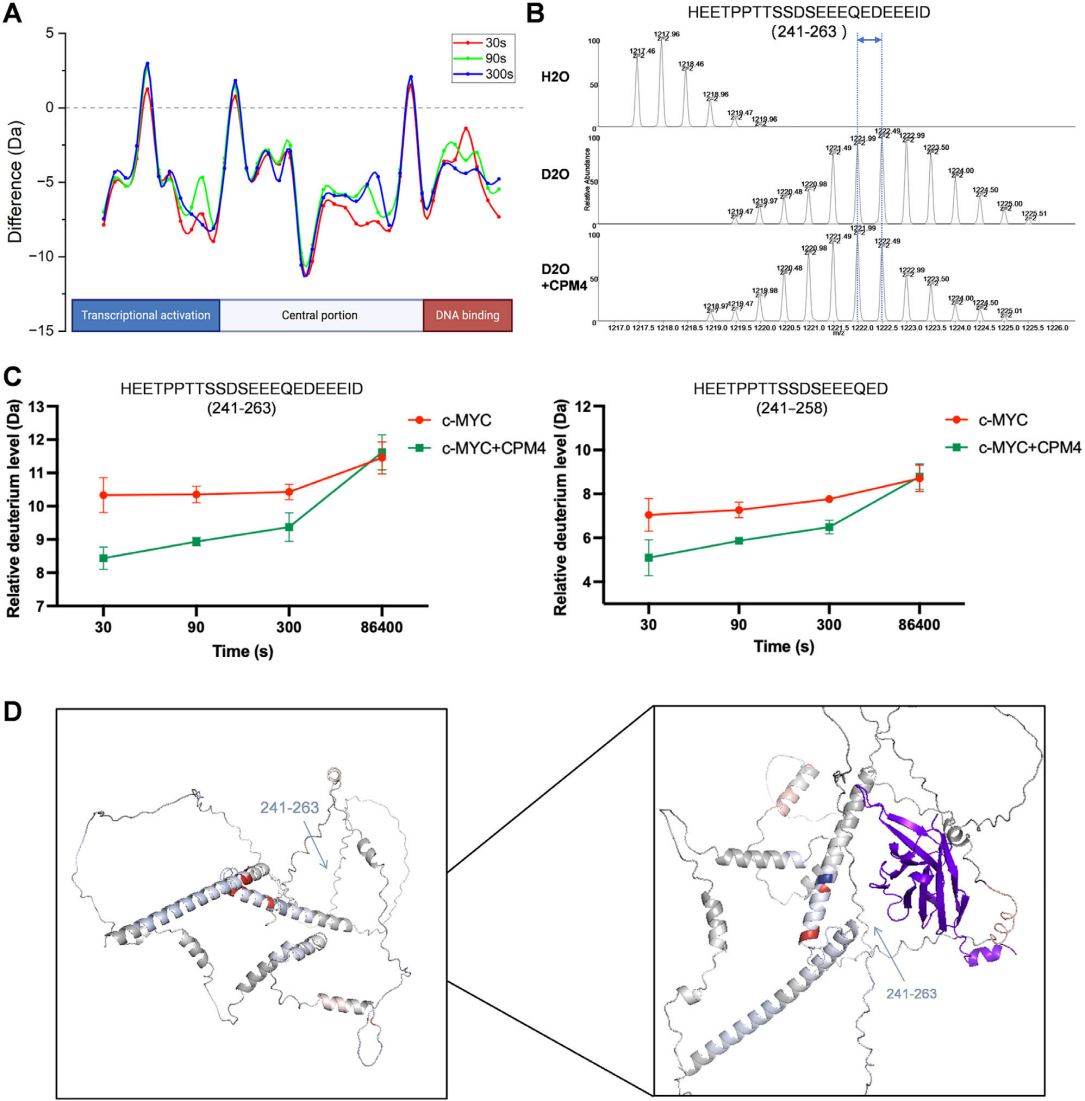
HDX‐MS mapping of the CPM4 epitope on c‐MYC. (A) Difference plot of hydrogen–deuterium exchange (HDX) for c‐MYC in the presence versus absence of CPM4 at the indicated labeling times (30, 90, and 300 s). Negative values indicate reduced deuterium uptake and increased protection upon CPM4 binding. Data from three independent experiments were obtained; one representative dataset is shown. (B) Representative mass spectra of a c‐MYC peptide from the HDX‐MS analysis. The top panel shows the undeuterated peptide, the middle panel shows the peptide after 30 s in D_2_O, and the bottom panel shows the same peptide after 30 s in D_2_O in the presence of CPM4. Dashed lines indicate the isotope envelope shift; reduced deuterium incorporation is observed upon CPM4 binding. (C) Time course of deuterium uptake for selected c‐MYC peptides in the absence (control) or presence of CPM4, illustrating decreased exchange kinetics upon nanobody binding. Values represent mean ± SD (*n* = 3). (D) Structural mapping of peptides showing significant changes in deuterium exchange onto the c‐MYC structure. Peptides with increased deuterium uptake are colored red, those with decreased uptake (protection) are colored blue, and CPM4‐interacting peptides within the epitope region are highlighted in purple.

To delineate the structural epitope on c‐MYC engaged by CPM4, we carried out hydrogen–deuterium exchange mass spectrometry (HDX‐MS) using recombinant c‐MYC in the absence or presence of CPM4 at the same molar ratio used in functional assays. Proteins were incubated in D2O for varying time intervals, quenched, digested, and analyzed by LC–MS to monitor deuterium incorporation across overlapping peptides. Comparison of deuterium uptake patterns between free c‐MYC and the CPM4–c‐MYC complex revealed several regions with markedly decreased exchange upon CPM4 binding, indicative of reduced local flexibility and solvent accessibility (Figure 3A). There was a marked decrease in deuterium uptake efficiency in peptides, primarily corresponding to residues 241–263 of c‐MYC after CPM4 treatment. This indicated that the mobility of these motifs was constrained by their interaction with CPM4 (Figure 3B).

With increasing labeling time, the CPM4‐associated protection from deuterium incorporation within the 241–263 segment of c‐MYC progressively weakened, and the largest decrease was observed at the 30‐s time point (Figure 3C). This indicates that CPM4 predominantly interacts with conformational epitopes within the 241–263 domain of c‐MYC. Notably, this domain lies within the central PEST sequence of c‐MYC (amino acids 226–270), a motif linked to rapid MYC turnover [50]. These observations raise the possibility that CPM4 engagement of the PEST region contributes to, or is coupled with, enhanced c‐MYC degradation. For structural interpretation, HDX‐MS protection patterns were projected onto the c‐MYC structural model to visualize the CPM4‐binding surfaces (Figure 3D). The results provide a detailed representation of the specific regions where CPM4 interacts with c‐MYC, highlighting its distinct binding characteristics.

### 2.4 CPM4 Induces Apoptosis and Impairs Motility of Tumor Cells

**FIGURE 4:**
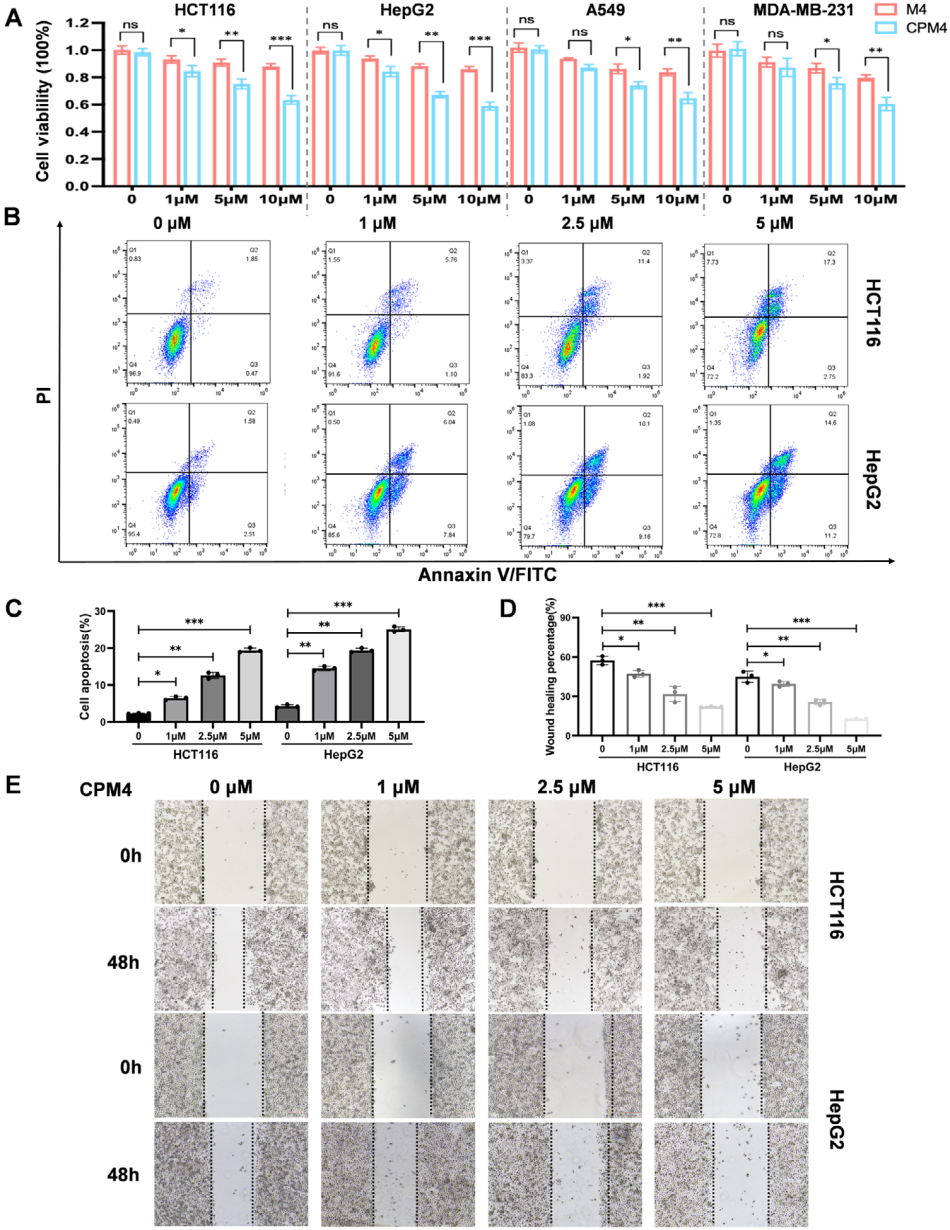
CPM4 reduces viability, induces apoptosis, and impairs migration of c‐MYC‐dependent cancer cells. (A) Cell viability of HCT116, HepG2, A549, and MDA‐MB‐231 cells after 24 h treatment with PBS, M4, or CPM4 at the indicated concentrations, measured by CCK‐8 assay and normalized to PBS controls. (B) Representative flow‐cytometric dot plots of Annexin V/PI staining in HCT116 and HepG2 cells treated with vehicle or CPM4 (1, 2.5, or 5 µM) for 24 h. (C) Quantification of total apoptotic cells (early + late apoptosis) from the experiments shown in (B). (D) Quantification of wound closure in scratch assays of HCT116 and HepG2 monolayers treated with vehicle or CPM4 (1, 2.5, or 5 µM) for 48 h. (E) Representative phase‐contrast images of scratch wounds in HCT116 and HepG2 monolayers at 0 and 48 h under the indicated CPM4 treatments, illustrating CPM4‐induced inhibition of cell migration. Data are presented as mean ± SEM (*n* = 3 biological replicates). *p* < 0.05; *p < 0.01; **p < 0.001 (Student's *t*‐test).

We next examined whether CPM4 elicits functional effects in c‐MYC‐dependent cancer cells. To this end, a panel of MYC‐driven cell lines—including HCT116 colorectal carcinoma, HepG2 hepatocellular carcinoma, A549 lung carcinoma, and MDA‐MB‐231 breast carcinoma cells—was treated with vehicle, unconjugated M4, CPP alone, or CPM4, and cell viability was quantified 24 h after treatment. M4 alone did not significantly impact cell viability in the absence of the CPP component (Figure 4A), and the CPP alone exhibited no notable biological activity (Figure S2A). In contrast, CPM4 caused a pronounced, dose‐dependent reduction in viable cell numbers across all three cell lines, consistent with effective inhibition of c‐MYC function and supporting its potential as an anticancer agent.

**FIGURE 5:**
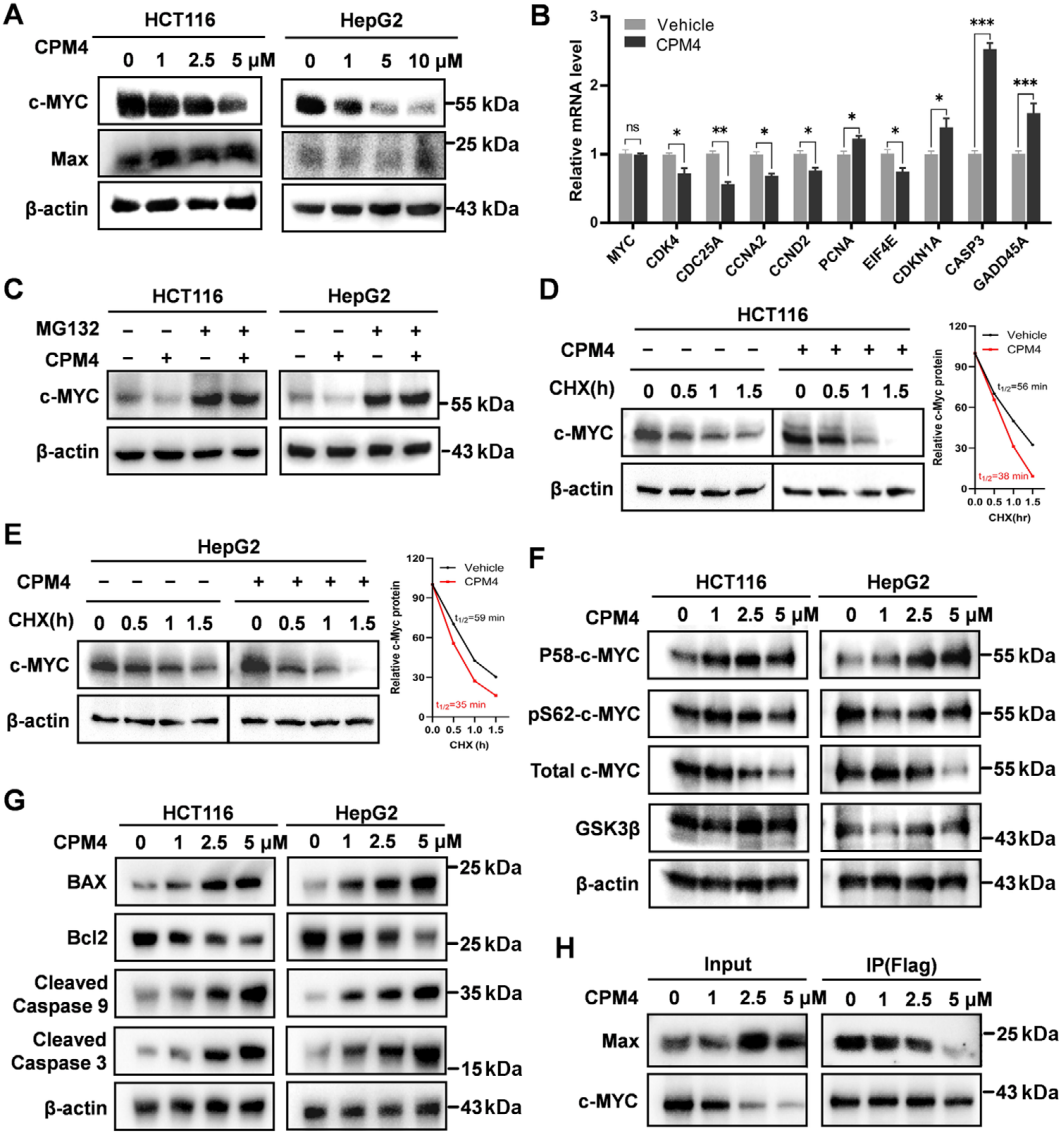
CPM4 promotes proteasomal degradation of c‐MYC and modulates downstream signaling. (A) Western blot analysis of c‐MYC and MAX in HCT116 and HepG2 cells treated with increasing concentrations of CPM4 for 24 h; β‐actin serves as a loading control. (B) Quantitative RT‐PCR analysis of selected c‐MYC target genes in HCT116 cells treated with CPM4, presented as relative mRNA levels normalized to vehicle control. (C) HCT116 and HepG2 cells were pretreated with MG132 (10 µM, 6 h) followed by CPM4 (5 µM, 24 h). c‐MYC protein levels were examined by Western blotting to assess proteasome dependence. (D and E) Cycloheximide (CHX) chase assays in HCT116 and HepG2 cells pretreated with vehicle or CPM4 for 3 h. Cells were harvested at the indicated times after CHX addition, and c‐MYC levels were analyzed by Western blotting; representative decay curves and calculated half‐lives are shown. (F) Western blot analysis of p‐c‐MYC (Thr58), p‐c‐MYC (Ser62), total c‐MYC, and GSK3β in HCT116 and HepG2 cells treated with CPM4 (1, 2.5, or 5 µM) for 24 h. β‐Actin is shown as a loading control. (G) Expression of apoptosis‐related proteins Bcl‐2, BAX, cleaved caspase‐3, and cleaved caspase‐9 in HCT116 and HepG2 cells treated with increasing concentrations of CPM4 for 24 h, assessed by Western blotting; β‐actin serves as a loading control. (H) Coimmunoprecipitation of c‐MYC and MAX in HCT116 cells with or without CPM4 treatment (1 h), showing CPM4‐induced disruption of the c‐MYC/MAX complex. Data are shown as mean ± SEM (*n* = 3); *p* < 0.05, *p < 0.01, **p < 0.001.

To determine whether the loss of viability was associated with apoptosis, we performed Annexin V/Alexa Fluor 488 and propidium iodide staining followed by flow cytometry. CPM4 treatment significantly increased the fraction of early and late apoptotic cells in HCT116, and vHepG2 cultures compared with vehicle or CPP controls (Figure 4B,C). Western blotting analysis confirmed that CPM4 activated apoptosis‐related markers, including Bcl‐2 and Bax, which are crucial components of the intrinsic apoptotic pathway involving caspases 3 and 9 (Figure 5E).

CPM4 also impaired the migratory behavior of tumor cells. In wound‐healing assays, CPM4‐treated monolayers exhibited markedly delayed closure of scratch gaps relative to PBS‐treated controls, and transwell migration assays confirmed a concentration‐dependent reduction in cell motility upon CPM4 exposure (Figure 4D,E). Together, these results demonstrate that CPM4 not only suppresses proliferation but also induces apoptosis and reduces migratory capacity in several c‐MYC‐expressing cancer cell lines.

### 2.5 CPM4 Decreases MYC Protein Stability by Enhancing Thr58 Phosphorylation

To elucidate the mechanism underlying the antiproliferative effects of CPM4, we first examined c‐MYC and MAX protein levels in HCT116 cells after CPM4 treatment. CPM4 reduced c‐MYC protein abundance in a dose‐dependent manner, whereas MAX levels remained essentially unchanged (Figure 5A). In parallel, c‐MYC mRNA levels were not significantly affected (Figure 5B), suggesting that CPM4 primarily regulates c‐MYC at the posttranscriptional level by altering protein stability rather than transcription.

To further address this, we employed the proteasome inhibitor MG132, which effectively prevented the CPM4‐induced decrease in c‐MYC protein, indicating that CPM4 promotes proteasome‐dependent c‐MYC degradation (Figure 5C). We next performed cycloheximide (CHX) chase assays to evaluate the impact of CPM4 on c‐MYC half‐life. CPM4 treatment markedly accelerated c‐MYC turnover, shortening its half‐life from approximately 56 to 38 min in HCT116 cells and from 59 to 35 min in HepG2 cells (Figure 5D,E). Collectively, these data demonstrate that CPM4 decreases c‐MYC stability and enhances its proteasomal degradation.

Extensive work has established that MYC stability is controlled by a hierarchical phosphorylation cascade, in which phosphorylation at Ser62 initially stabilizes the protein, whereas subsequent phosphorylation at Thr58 promotes recognition by E3 ubiquitin ligases and targeting to the proteasome. We therefore examined whether CPM4 affects these regulatory phosphorylation events. CPM4 increased Thr58 phosphorylation on c‐MYC in a dose‐dependent manner, without appreciably altering Ser62 phosphorylation or GSK3β levels (Figure 5F). These findings indicate that CPM4 primarily drives MYC degradation by enhancing Thr58 phosphorylation. Together with our epitope‐mapping data, they support a model in which CPM4 binding to the PEST region facilitates or stabilizes the Thr58‐phosphorylated state, thereby favoring ubiquitin‐mediated degradation and accelerating c‐MYC turnover.

Moreover, CPM4 also impacted apoptosis‐associated signaling. In both HCT116 and HepG2 cells, Bcl‐2 expression was markedly downregulated, whereas BAX, cleaved caspase‐3, and cleaved caspase‐9 were upregulated in a dose‐dependent manner (Figure 5G), consistent with activation of intrinsic apoptotic pathways. In addition, FLAG‐based affinity purification revealed that CPM4 impaired formation of the c‐MYC/MAX heterodimer (Figure 5H), which in turn reduced binding of the c‐MYC/MAX complex to an E‐box motif probe (Figure S2B). Functionally, CPM4 attenuated transcriptional activation of canonical c‐MYC downstream targets, leading to reduced expression of c‐MYC induced genes and upregulation of c‐MYC repressed genes in HCT116 cells (Figure 5B). These findings highlight the dual pathways through which CPM4 inhibits the oncogenic function of c‐MYC, and its potential as an effective therapeutic agent for c‐MYC‐driven cancers.

### 2.6 CPM4 Downregulates c‐MYC‐Targeted Downstream Genes

**FIGURE 6:**
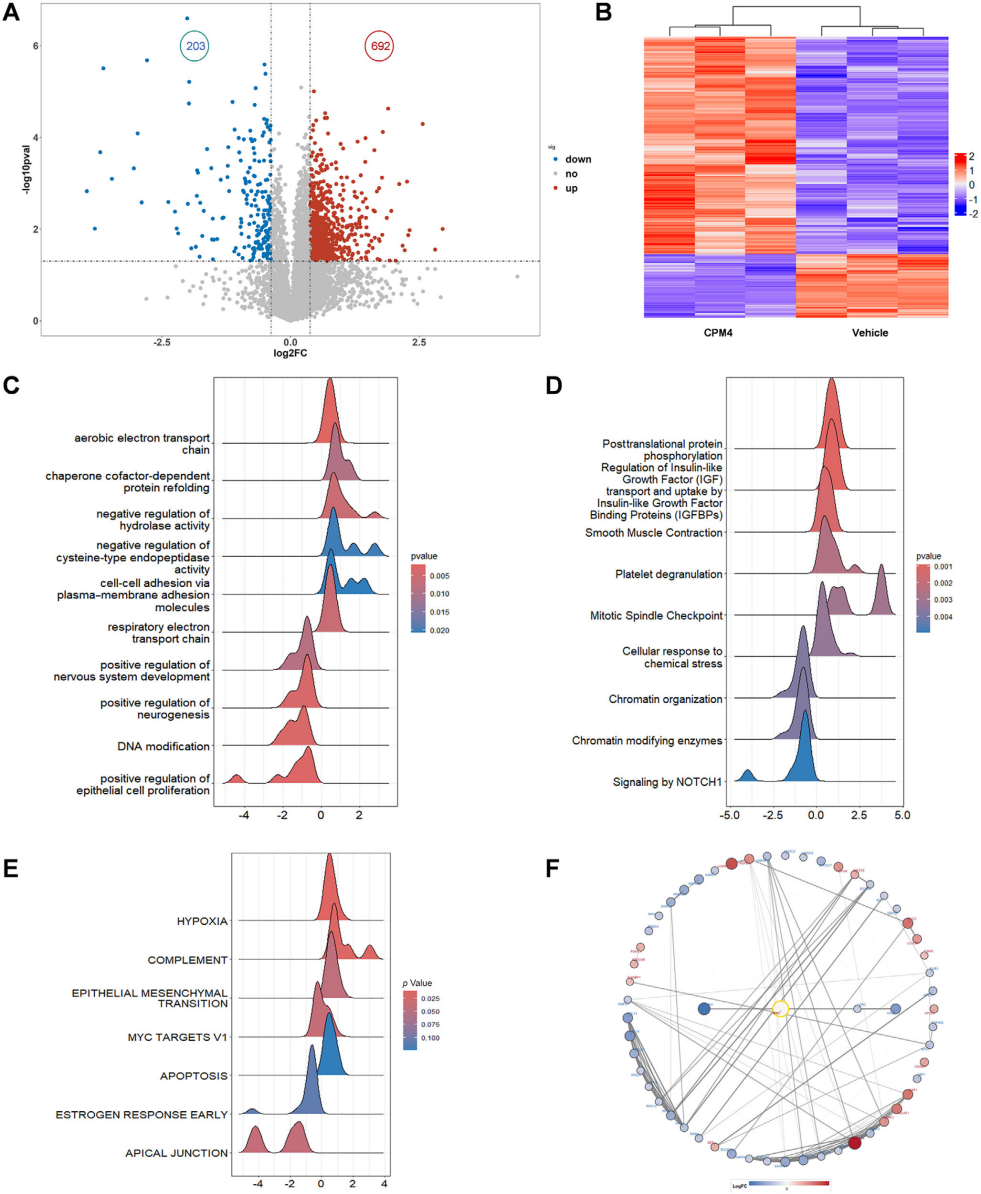
Proteomic analysis of CPM4‐treated HCT116 cells. (A) Volcano plot of differentially expressed proteins in CPM4‐treated versus vehicle‐treated HCT116 cells. Significantly upregulated and downregulated proteins (|log_2_ fold change| > 0.5, *p* < 0.05) are shown in red and blue, respectively; nonsignificant proteins are shown in gray. (B) Heatmap of significantly altered proteins clustered by unsupervised hierarchical clustering. Columns represent biological replicates of each treatment group and rows represent individual proteins; values are displayed as *z*‐scored protein abundance. (C and D) Gene set enrichment analysis (GSEA) of CPM4‐induced proteomic changes. Proteins were ranked by log_2_ fold change, and enrichment was assessed for selected GO Biological Process (GOBP) terms (C) and Reactome pathways (D). Ridge plots depict the distribution of ranked proteins contributing to each term, with color intensity indicating nominal p values. (E) GSEA of selected Hallmark gene sets at the protein level, including HALLMARK_MYC_TARGETS_V1. Negative enrichment of MYC target signatures in CPM4‐treated cells is consistent with CPM4‐mediated repression of MYC‐dependent programs. (F) Protein–protein interaction (PPI) network of significantly altered proteins overlapping with the HALLMARK_MYC_TARGETS_V1 gene set, constructed using the STRING database (high‐confidence interactions). The network highlights CPM4‐regulated clusters of c‐MYC downstream and associated proteins. Node color indicates log_2_ fold change (red, upregulated; blue, downregulated), and node size reflects connectivity (degree).

Because c‐MYC functions as a global transcriptional regulator, we next investigated how CPM4‐induced depletion of c‐MYC reshapes downstream protein networks. To this end, we performed quantitative proteomic profiling of HCT116 cells treated with vehicle or CPM4. In total, 895 proteins were significantly altered upon CPM4 treatment, including 692 upregulated and 203 downregulated species (|log_2_ fold change| > 0.5, *p* < 0.05; Figure 6A). Unsupervised hierarchical clustering of these differentially expressed proteins clearly segregated CPM4‐treated samples from controls (Figure 6B), indicating a coherent, treatment‐dependent remodeling of the proteome. Notably, many of the downregulated proteins correspond to established c‐MYC‐regulated factors involved in cell‐cycle progression, DNA replication, ribosome biogenesis, and intermediary metabolism, consistent with inhibition of MYC‐driven programs.

To obtain a pathway‐level view, we applied gene set enrichment analysis (GSEA) to the ranked protein list. GSEA of Gene Ontology Biological Process terms revealed negative enrichment of pathways related to cell‐cycle regulation, mitotic spindle checkpoint control, chromatin organization and modification, and DNA‐associated processes, whereas terms linked to aerobic electron transport, stress responses, and protein quality control, including chaperone‐mediated protein refolding and ubiquitin‐related functions, were positively enriched in CPM4‐treated cells (Figure 6C). Reactome pathway GSEA further highlighted suppression of signaling modules associated with proliferation and cell‐cycle progression, together with activation of pathways related to cellular stress and chromatin remodeling (Figure 6D). These data indicate that CPM4 does not merely reduce the abundance of individual c‐MYC targets but also perturbs coordinated protein networks that support rapid cell division and genomic integrity.

We then examined Hallmark gene sets at the protein level. CPM4 treatment significantly affected several cancer‐relevant signatures, including epithelial–mesenchymal transition, apoptosis, hypoxia, and, importantly, HALLMARK_MYC_TARGETS_V1, which exhibited clear negative enrichment (Figure 6E). The depletion of MYC target signatures at the proteomic level is in line with the observed decrease in c‐MYC protein and transcriptional activity and provides independent systems‐level support for CPM4‐mediated repression of MYC‐dependent oncogenic programs.

Finally, we constructed a protein–protein interaction (PPI) network from significantly differentially expressed proteins that overlapped with the HALLMARK_MYC_TARGETS_V1 gene set using the STRING database. The resulting network revealed interconnected clusters of c‐MYC downstream and associated proteins involved in proliferation, DNA damage responses, chromatin regulation, and metabolic control that were markedly modulated by CPM4 (Figure 6F). Together, these proteomic and network analyses demonstrate that CPM4 treatment induces a coordinated reprogramming of MYC‐associated protein networks, providing a mechanistic framework for its antiproliferative, proapoptotic, and antimigratory effects in c‐MYC‐dependent cancer cells.

### 2.7 CPM4 Suppresses MYC‐Driven Tumorigenesis in Vivo

**FIGURE 7:**
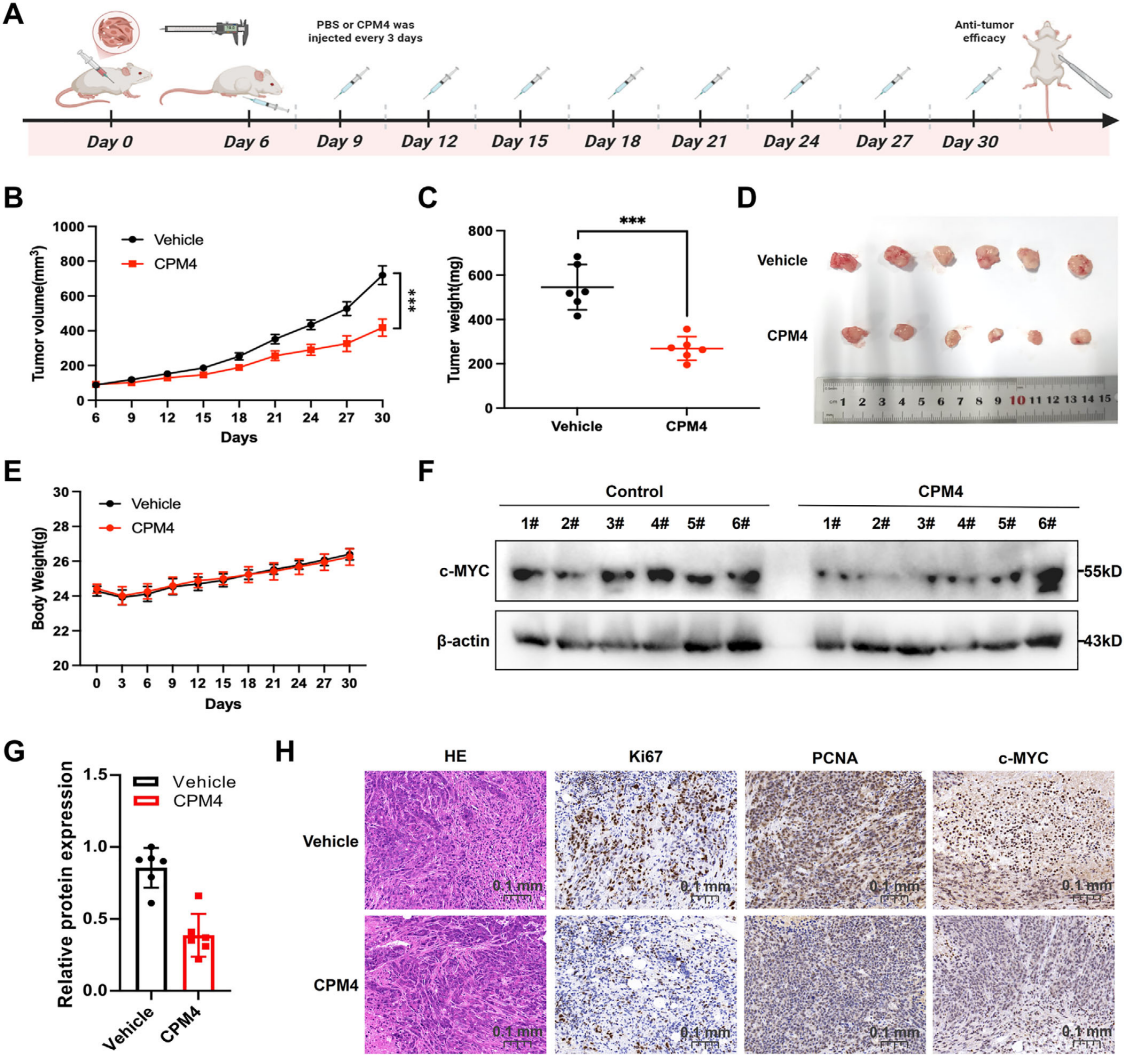
In vivo antitumor efficacy of CPM4 in a HCT116 xenograft model. (A) Schematic representation of the experimental timeline and dosing regimen. Mice bearing subcutaneous HCT116 tumors received vehicle (PBS) or CPM4 every 3 days, and tumor volume was measured throughout the treatment period. (B) Tumor growth curves for vehicle‐ and CPM4‐treated groups, with tumor volume monitored every 3 days. (C) Endpoint tumor weights in vehicle‐ and CPM4‐treated mice. (D) Representative photographs of excised tumors from each group, illustrating the reduction in tumor size after CPM4 treatment. (E) Terminal body weight comparison across treatment groups. (F) Immunoblot analysis of c‐MYC expression in tumor lysates from vehicle‐ and CPM4‐treated mice; β‐actin was used as a loading control. (G) Densitometric quantification of c‐MYC protein levels normalized to β‐actin. (H) Representative H&E staining and immunohistochemistry for Ki‐67, PCNA, and c‐MYC in xenograft sections from vehicle‐ and CPM4‐treated mice. Scale bars, 100 µm. Data are presented as mean ± SEM. *p* < 0.05; *p < 0.01; **p < 0.001.

The in vivo antitumor efficacy of CPM4 was systematically assessed using an established HCT116 xenograft model in NSG mice. Following successful tumor engraftment, animals were randomly assigned to receive either PBS or CPM4 (10 mg/kg) via intraperitoneal injection every 3 days (Figure 7A). After 30 days of treatment, quantitative analysis revealed that CPM4 administration significantly and dose dependently inhibited tumor growth compared with the vehicle control (Figure 7B). Tumor growth curves showed markedly slower progression in CPM4‐treated mice, and endpoint measurements confirmed substantial reductions in both tumor volume and tumor weight (Figure 7C,D). Notably, CPM4 treatment was well tolerated, with no significant changes in body weight or other clinical signs of toxicity observed during the experimental period (Figure 7E). In a separate tolerability cohort of healthy mice, the same dosing regimen of CPM4 did not induce apparent histopathological abnormalities in the heart, liver, spleen, lung, kidney, or small intestine (Figure S3).

At the molecular level, CPM4 treatment significantly modulated key oncogenic markers within tumor tissues. Western blot analysis of tumor lysates revealed a pronounced downregulation of c‐MYC protein expression (Figure 7F,G). Histopathological evaluation, including hematoxylin and eosin staining, revealed extensive tumor necrosis and structural disruption in CPM4‐treated specimens. Immunohistochemical (IHC) analysis further confirmed robust suppression of c‐MYC expression and significant reductions in proliferation markers Ki67 and PCNA in the CPM4‐treated group (Figure 7H). Collectively, these findings establish CPM4 as a potent and selective inhibitor of c‐MYC‐driven tumorigenesis, offering a promising therapeutic window for further clinical development.

## 3 Discussion

In this study, we show that engineered c‐MYC‐specific nanobodies, in particular the M4 clone and its cell‐permeable derivative CPM4, can be used to functionally silence the oncogenic transcription factor c‐MYC in cancer cells. c‐MYC is a central driver of malignant transformation but has long resisted conventional drug discovery because of its largely disordered structure and lack of canonical ligandable pockets [2]. Here, we demonstrate that M4 recognizes a defined region within the PEST domain (amino acids 241–263), enhances Thr58 phosphorylation, and promotes proteasome‐dependent degradation of c‐MYC. At the same time, CPM4 interferes with c‐MYC/MAX heterodimer formation and attenuates MYC‐dependent transcriptional programs. This combination of enforced protein destabilization and transcriptional inhibition results in robust suppression of MYC‐driven oncogenic pathways. Consistent with these molecular effects, CPM4 reduces tumor growth in xenograft models, supporting its potential as a targeted biologic for MYC‐addicted malignancies. Notably, CPM4 complements existing MYC‐targeting efforts that mainly aim to inhibit MYC/MAX function without necessarily depleting MYC protein and may therefore offer an additional layer of suppression through oncoprotein reduction. Among direct MYC inhibitors, the dominant‐negative Omomyc program has recently achieved clinical translation (OMO‐103, Phase I), supporting the feasibility of directly targeting MYC in patients and providing an important benchmark for future MYC‐directed biologics [51]. Compared with small‐molecule MYC/MAX disruptors such as 10058‐F4, which suffers from weak binding affinity and limited in vivo efficacy, and MYCi975, which promotes MYC degradation via a distinct pharmacological mechanism targeting the MYC/MAX interface, CPM4 achieves epitope‐selective engagement of the PEST region with nanomolar affinity and dual functionality [52, 53]. Indirect strategies, most notably BET bromodomain inhibitors (e.g., JQ1), suppress MYC at the transcriptional level but frequently encounter resistance through BRD4‐independent MYC reactivation [54]. PROTAC‐based approaches, while conceptually appealing, remain constrained by the lack of a suitable ligandable handle on intrinsically disordered targets such as MYC [55]. CPM4 circumvents these limitations by directly binding a defined epitope and harnessing endogenous degradation machinery without requiring an exogenous E3 ligase‐recruiting moiety.

These findings add to a growing body of work establishing nanobodies as versatile tools for interrogating and modulating proteins that are difficult to target with small molecules or full‐length antibodies. The compact size, stability, and ability of nanobodies to recognize cryptic or flexible epitopes make them well suited for engaging intrinsically disordered regions and PPIs, such as those that govern c‐MYC activity. It is also worthwhile to compare the cell‐permeable MYC nanobody named CPMycNB developed in our previous study, with CPM4 [39]. CPMycNB was designed based on a nanobody identified by the Cambridge group and was subsequently conjugated with a cell‐permeable peptide [56]. On the contrary, CPM4 was developed based on a nanobody identified from our phage‐display screening. These two nanobodies have different sequences. Second, CPMycNB disrupts c‐MYC/MAX complex formation to repress MYC‐driven transcription. By contrast, CPM4 binds the PEST region of MYC and promotes its Thr58 phosphorylation and proteasome‐dependent degradation. Thus, CPM4 represents a mechanistically and functionally distinct modality from CPMycNB, extending nanobody‐based MYC targeting from transient functional blockade toward robust oncoprotein depletion with enhanced translational potential. Mechanistically, this degradation‐oriented effect is consistent with prior work showing that (i) a PEST sequence in c‐MYC (reported around amino acids 226–270) is linked to rapid MYC turnover and (ii) Thr58‐centered phosphodegron signaling promotes recognition by the SCFFbw7 ubiquitin ligase and proteasomal degradation [57]. Therefore, CPM4 binding near the PEST‐containing region may plausibly bias MYC toward a degradation‐competent state by increasing local accessibility for phosphorylation and/or downstream degron recognition, while also being compatible with parallel interference of MYC/MAX assembly. An intriguing and unexpected observation is that a single nanobody targeting the PEST domain can simultaneously promote proteasomal degradation and disrupt MYC/MAX dimerization‐two activities that are not a priori predicted to coexist within one binding modality. This dual functionality may reflect an allosteric coupling between PEST‐region engagement and conformational changes at the MYC/MAX interface, a hypothesis that warrants further investigation through high‐resolution structural analyses. In this regard, CPM4 differs from recently described intrabody‐degron tag fusions, which require an exogenous degradation signal to deplete target proteins [58]; instead, CPM4 leverages a native phosphodegron to trigger endogenous turnover.

Despite the promising activity of CPM4, several challenges must be addressed before clinical translation can be considered. Although we observe efficient intracellular delivery and antitumor effects in vitro and in xenograft models, the pharmacokinetic behavior, tissue distribution, and long‐term safety profile of CPM4 in more complex physiological settings remain to be defined. As nanobodies are generally cleared rapidly via renal filtration due to their small size, half‐life extension strategies such as PEGylation, albumin‐binding domains, or Fc fusion may be necessary to achieve sustained tumor exposure [59]. The use of a generic CPP, while enabling nuclear entry, raises concerns about off‐tumor uptake and potential immune responses. These issues highlight the need for improved delivery strategies, such as nanoparticle‐based carriers, tumor‐targeted CPP variants, or ligand‐guided delivery systems that enrich CPM4 in MYC‐driven tumors while limiting systemic exposure. Furthermore, tumor cells may adapt by altering the M4‐binding epitope, modulating MYC turnover pathways (e.g., downregulating Fbw7), or activating compensatory signaling networks (e.g., MYCN or MYCL upregulation), which could give rise to acquired resistance [57]. Systematic analysis of such adaptive mechanisms will be important for anticipating and counteracting resistance in future applications. An additional point is that CPP‐mediated intracellular delivery can be context dependent (cell type, serum, endosomal escape), and optimizing delivery/escape chemistry may be important for maximizing nuclear target engagement while minimizing nonspecific distribution [60]. Finally, the current study relies on immunodeficient xenograft models, which do not fully recapitulate the host immune microenvironment; future evaluation in syngeneic or humanized mouse models will be essential to assess immune‐related effects and potential immunogenicity of CPM4.

Overall, our data establish CPM4 as a promising candidate for therapeutic intervention in c‐MYC‐dependent cancers. By coupling promotion of c‐MYC degradation with suppression of its transcriptional activity, CPM4 exemplifies a multilayered strategy to neutralize an oncogene long considered intractable to pharmacologic targeting. Future work should extend preclinical evaluation to immunocompetent and patient‐derived xenograft models to assess efficacy across diverse tumor microenvironments, refine delivery platforms to enhance tumor selectivity and minimize off‐target effects, and systematically explore combination regimens with chemotherapy, kinase inhibitors, or immunotherapies to identify synergistic interactions and delay resistance. High‐resolution structural studies of the M4–c‐MYC complex (e.g., by cryo‐EM or X‐ray crystallography) could further guide affinity maturation and epitope engineering, enabling the rational design of next‐generation nanobodies with improved potency, selectivity, and developability for targeting c‐MYC and other undruggable oncoproteins. More broadly, CPM4 supports a generalizable blueprint for intracellular nanobody‐based degradation strategies: epitope‐selective recognition paired with programmable delivery and turnover control, which could be extended to other disordered or interface‐dominated oncoproteins that remain difficult to drug with small molecules.

## 4 Materials and Methods

### 4.1 Recombinant c‐MYC Protein Production

Full‐length human c‐MYC fused to a C‐terminal hexahistidine tag was expressed in *Escherichia coli* using the pET28a vector system. The recombinant protein accumulated in inclusion bodies, which were solubilized under denaturing conditions (8 M urea) and subsequently refolded by gradual dilution into nondenaturing buffer. The refolded protein was captured by immobilized metal affinity chromatography using Ni‐NTA resin, followed by size‐exclusion chromatography on a HiLoad 16/600 Superdex 200 column (Cytiva) to achieve high purity. Purified c‐MYC was concentrated, flash‐frozen, and stored at −80°C for subsequent phage display selections and binding studies. Detailed buffer compositions and purification parameters are provided in Supporting Information: Methods.

### 4.2 Construction of a Synthetic Nanobody Library

A fully synthetic VHH library was constructed based on a consensus framework sequence derived from camelid heavy‐chain antibodies. Sequence diversity was introduced into the three CDRs (CDR1, CDR2, and CDR3) using NNK degenerate codons through a multistep overlap‐extension PCR strategy. The assembled VHH genes were cloned into the pCantab 5E phagemid vector and introduced into *E. coli* TG1 by electroporation. Library size was estimated by serial dilution plating, and sequence diversity was confirmed by Sanger sequencing of randomly selected clones. Complete details of the PCR primers and cloning strategy are described in Supporting Information: Methods.

### 4.3 Phage Display Biopanning

c‐MYC‐specific nanobodies were enriched through four iterative rounds of phage display selection. Immunotubes coated with purified c‐MYC protein (50 µg/mL) served as positive selection surfaces, while BSA‐coated tubes were used as negative controls. After blocking and incubation with the phage library, unbound phages were removed by stringent washing, and specifically bound phages were eluted under alkaline conditions. Eluted phages were amplified in *E. coli* TG1 with M13KO7 helper phage assistance. Selection stringency was progressively increased across successive rounds to enrich high‐affinity binders. The complete panning protocol is provided in Supporting Information: Methods.

### 4.4 Screening for c‐MYC‐Reactive Clones

Individual clones from the final selection round were screened by phage ELISA. Monoclonal phage supernatants were incubated in microtiter plates coated with c‐MYC antigen or BSA control. Bound phages were detected using HRP‐conjugated anti‐M13 antibodies and TMB substrate development. Clones exhibiting signal‐to‐background ratios exceeding 2.0 were scored as positive binders and selected for further characterization.

### 4.5 Nanobody Expression and Purification

His‐tagged nanobodies were produced in *E. coli* BL21(DE3) using the pET‐21b expression system. Following IPTG induction, cells were harvested and lysed by sonication. Recombinant nanobodies were purified by Ni‐NTA affinity chromatography followed by size‐exclusion chromatography on a Superdex 75 Increase 10/300 GL column (Cytiva). Purified proteins were quantified spectrophotometrically, aliquoted, and stored at −80°C. Detailed expression and purification conditions are provided in Supporting Information: Methods.

### 4.6 Nanobody–CPP Conjugation

Cell‐permeable CPM4 was generated by Sortase A‐mediated transpeptidation to conjugate nanobody–LPETG–6×His (20 µM) to an N‐terminal GGG‐CPP peptide (50 µM) in Tris–HCl/NaCl/CaCl_2_ reaction buffer (30°C, 16 h). His‐tagged Sortase A and unreacted nanobody were removed by Ni‐NTA, and excess free CPP was removed by repeated ultrafiltration (10 kDa MWCO). Product identity and purity were assessed by SDS‐PAGE and MALDI–TOF–MS. CPP information, reaction composition, and batch acceptance criteria are provided in Table S4.

Site‐specific conjugation of nanobodies to CPPs was achieved through Sortase A‐mediated transpeptidation. Nanobodies engineered with a C‐terminal LPETG‐His_6_ motif were reacted with synthetic GGG‐CPP peptide (Bankpeptide Biological Technology) in the presence of recombinant Sortase A. Unreacted His‐tagged components were removed by subtractive Ni‐NTA chromatography, and excess peptide was eliminated by ultrafiltration. Conjugate integrity and purity were verified by MALDI–TOF–MS. Reaction conditions are detailed in Supporting Information: Methods.

### 4.7 Surface Plasmon Resonance Binding Analysis

Binding kinetics between c‐MYC and nanobodies were characterized by SPR using a Biacore 3000 instrument. Recombinant c‐MYC was immobilized on CM5 sensor chips via amine coupling chemistry to achieve approximately 1400 response units. Nanobody analytes were injected as a twofold dilution series (1–50 nM) at 25°C with a flow rate of 20 µL/min. Sensorgrams were globally fitted to a 1:1 Langmuir interaction model using BIAevaluation software to derive association rate constants (*k*_a_), dissociation rate constants (*k*_d_), and equilibrium dissociation constants (*K*_D_). Detailed SPR parameters are provided in Supporting Information: Methods.

### 4.8 Confocal Fluorescence Microscopy

HCT116 cells treated with M4 or CPM4 (10 µM, 24 h) were fixed, permeabilized, and immunostained with antibodies against the HA epitope tag and c‐MYC protein. Fluorophore‐conjugated secondary antibodies and DAPI nuclear counterstain were applied, and images were acquired using a Nikon A1R HD25 confocal microscope equipped with a 100× oil‐immersion objective. Complete staining protocols and antibody information are provided in Supporting Information: Methods and Table S1.

### 4.9 Hydrogen–Deuterium Exchange Mass Spectrometry

HDX‐MS experiments were performed to map PPI interfaces. Protein samples were incubated in D_2_O‐based labeling buffer, and exchange reactions were quenched under acidic reducing conditions. Samples were digested with pepsin and analyzed by LC–MS/MS using a Q Exactive mass spectrometer (Thermo Scientific). Deuterium incorporation was quantified using HDExaminer software by comparing centroid masses of deuterated versus nondeuterated peptide species. Detailed HDX‐MS parameters are described in Supporting Information: Methods.

### 4.10 Cell Culture

HCT116 and A549 cells were maintained in RPMI‐1640 medium; HepG2, MDA‐MB‐231, and HEK293T cells were cultured in DMEM. All media were supplemented with 10% fetal bovine serum and penicillin–streptomycin. Cells were incubated at 37°C in a humidified atmosphere containing 5% CO_2_. All cell lines were obtained from ATCC and routinely tested for mycoplasma contamination.

### 4.11 Immunoblot Analysis

Following treatment, cells were lysed in SDS‐containing buffer supplemented with protease inhibitors. Equal amounts of protein were resolved by SDS‐PAGE, transferred to PVDF membranes, and probed with the indicated primary antibodies. Protein bands were detected using HRP‐conjugated secondary antibodies and enhanced chemiluminescence. Complete antibody information is provided in Table S1.

### 4.12 Cell Viability and Apoptosis Assays

Cell viability was assessed using the CCK‐8 colorimetric assay (KeyGEN BioTECH). Cells seeded in 96‐well plates were treated with indicated concentrations of M4 or CPM4, and absorbance at 450 nm was measured following CCK‐8 reagent addition. Apoptosis was quantified by Annexin V‐APC/7‐AAD dual staining followed by flow cytometric analysis (BD Biosciences). Detailed protocols are provided in Supporting Information: Methods.

### 4.13 Xenograft Tumor Studies

All animal procedures were approved by the Institutional Animal Care and Use Committee of Tsinghua University. HCT116 cells (1 × 10^7^) were implanted subcutaneously into 6‐week‐old NSG mice. When tumors reached approximately 100 mm^3^, mice were randomized into treatment (CPM4, 10 mg/kg, i.p., every other day) or vehicle control groups (*n* = 6 per group). Tumor dimensions and body weight were recorded every 3 days throughout the 24‐day treatment period. Tumor volume was calculated as ½ × length × width^2^. At endpoint, tumors were excised for weight measurement and IHC analysis.

### 4.14 Immunohistochemistry

Formalin‐fixed, paraffin‐embedded tumor sections were subjected to heat‐induced antigen retrieval and immunostained with antibodies against c‐MYC, PCNA, or Ki‐67. Immunoreactivity was visualized using HRP‐conjugated secondary antibodies and DAB chromogen, with hematoxylin counterstaining. Antibody dilutions and staining protocols are detailed in Supporting Information: Methods.

## Author Contributions

Yuanyuan Xue conceptualized and supervised the project, designed the experimental framework, performed data analyses, and drafted the manuscript. Hao Jiang, Zhaoyun Zong, Xiaolin Tian, and Ting Li performed the experiments and contributed to data acquisition and analysis. Zelong Miao and Yali Wei provided substantial support in data processing and interpretation. Haiteng Deng contributed advanced data analysis and critically revised the manuscript to ensure intellectual rigor and scientific accuracy. All authors have read and approved the final manuscript.

## Funding Information

This work was supported by the National Key Research and Development Program of China (Grant No. 2021YFA1302601), the National Natural Science Foundation of China (Grant Nos. 82172556 and T2293763), the Beijing Natural Science Foundation (Grant No. IS24040), and the Open Research Fund of the State Key Laboratory of Complex, Severe, and Rare Diseases (No. 2025‐I‐PY‐001).

## Ethics Statement

All animal experiments in this study were performed in accordance with institutional guidelines and were approved by the Laboratory Animal Use and Management Committee of the Laboratory Animal Center of Tsinghua University (welfare ethics review approval No. 333, 2025).

## Conflicts of Interest

There are no conflicts to declare.

## Supporting information




**Supporting Figure 1**: Purification and Characterization of Nanobodies.(A‐F) Size‐exclusion chromatography and SDS‐PAGE analysis of purified nanobodies, including M4,M10, M14, M41, Sortase A and MAX. The chromatograms and gels demonstrate the purity and integrity of the nanobodies.(G) Western blot analysis showing the results of a pull‐down assay where c‐MYC was extracted from 293T cell lysate using either purified nanobodies or no nanobodies (negative control). The blot confirms the presence of c‐MYC specifically pulled down by the nanobodies.
**Supporting Figure 2**: Analysis of M4 and CPM4 Effects. (A) Cell viability of HCT116, HepG2, A549, and MDA‐MB‐231 cells treated with CPP, with PBS vehicle as the control. (B) c‐MYC and MAX were incubated with various concentrations of CPM4, followed by addition of a biotin‐labeled E‐box probe for electrophoretic mobility shift assay (EMSA). (C) MALDI–TOF–mass spectra confirming conjugation of M4 with a CPP.
**Supporting Figure 3**: Histopathological assessment of major organs following CPM4 administration. Representative hematoxylin and eososin (H&E)‐stained sections of heart, liver, spleen, lung, kidney, and small intestine collected from mice treated with vehicle (PBS) or CPM4 (treatment regimen as described in Methods). Scale bar, 0.1 mm.
**Supporting Table 1**: Antibodies used in this study.

## Data Availability

All raw mass spectrometry data have been deposited in the iProX repository and are publicly available under the accession number IPX0014717001.

## References

[1] F. Bray , M. Laversanne , H. Sung , et al., “Global Cancer Statistics 2022: GLOBOCAN Estimates of Incidence and Mortality Worldwide for 36 Cancers in 185 Countries,” CA: A Cancer Journal for Clinicians 74 (2024): 229–263. 38572751 10.3322/caac.21834

[2] C. V. Dang , “MYC on the Path to Cancer,” Cell 149, no. 1 (2012): 22–35. 22464321 10.1016/j.cell.2012.03.003 PMC3345192

[3] S. C. Casey , V. Baylot , and D. W. Felsher , “The MYC Oncogene Is a Global Regulator of the Immune Response,” Blood 131, no. 18 (2018): 2007–2015. 29514782 10.1182/blood-2017-11-742577 PMC5934797

[4] J. A. Nilsson and J. L. Cleveland , “Myc Pathways Provoking Cell Suicide and Cancer,” Oncogene 22, no. 56 (2003): 9007–9021. 14663479 10.1038/sj.onc.1207261

[5] A. Papadimitropoulou , M. Makri , and G. Zoidis , “MYC the Oncogene From Hell: Novel Opportunities for Cancer Therapy,” European Journal of Medicinal Chemistry 267 (2024): 116194. 38340508 10.1016/j.ejmech.2024.116194

[6] C. V. Dang , L. M. Resar , E. Emison , et al., “Function of the c‐Myc Oncogenic Transcription Factor,” Experimental Cell Research 253, no. 1 (1999): 63–77. 10579912 10.1006/excr.1999.4686

[7] C. Y. Lin , J. Lovén , P. B. Rahl , et al., “Transcriptional Amplification in Tumor Cells With Elevated c‐Myc,” Cell 151, no. 1 (2012): 56–67. 23021215 10.1016/j.cell.2012.08.026 PMC3462372

[8] C. Lourenco , D. Resetca , C. Redel , et al., “MYC Protein Interactors in Gene Transcription and Cancer,” Nature Reviews Cancer 21, no. 9 (2021): 579–591. 34188192 10.1038/s41568-021-00367-9

[9] C. J. Poole and J. van Riggelen , “MYC—master Regulator of the Cancer Epigenome and Transcriptome,” Genes (Basel) 8, no. 6 (2017): 142. 28505071 10.3390/genes8050142 PMC5448016

[10] C. Sequera , M. Grattarola , A. Holczbauer , et al., “MYC and MET Cooperatively Drive Hepatocellular Carcinoma With Distinct Molecular Traits and Vulnerabilities,” Cell Death & Disease 13, no. 11 (2022): 994. 36433941 10.1038/s41419-022-05411-6 PMC9700715

[11] L. Nguyen , P. Papenhausen , and H. Shao , “The Role of c‐MYC in B‐cell Lymphomas: Diagnostic and Molecular Aspects,” Genes (Basel) 8, no. 4 (2017): 116. 28379189 10.3390/genes8040116 PMC5406863

[12] J. Xu , Y. Chen , and O. I. Olopade , “MYC and Breast Cancer,” Genes & Cancer 1, no. 6 (2010): 629–640. 21779462 10.1177/1947601910378691 PMC3092228

[13] L. Tan , D. Peng , and Y. Cheng , “Significant Position of C‐myc in Colorectal Cancer: A Promising Therapeutic Target,” Clinical & Translational Oncology 24, no. 12 (2022): 2295–2304. 35972682 10.1007/s12094-022-02910-y

[14] R. Dhanasekaran , A. S. Hansen , J. Park , et al., “MYC Overexpression Drives Immune Evasion in Hepatocellular Carcinoma That Is Reversible Through Restoration of Proinflammatory Macrophages,” Cancer Research 83, no. 4 (2023): 626–640. 36525476 10.1158/0008-5472.CAN-22-0232 PMC9931653

[15] C. V. Dang , A. Le , and P. Gao , “MYC‐Induced Cancer Cell Energy Metabolism and Therapeutic Opportunities,” Clinical Cancer Research 15, no. 21 (2009): 6479–6483. 19861459 10.1158/1078-0432.CCR-09-0889 PMC2783410

[16] Z. E. Stine , Z. E. Walton , B. J. Altman , A. L. Hsieh , and C. V. Dang , “MYC, Metabolism, and Cancer,” Cancer Discovery 5, no. 10 (2015): 1024–1039. 26382145 10.1158/2159-8290.CD-15-0507 PMC4592441

[17] T. Wahlström and M. Arsenian Henriksson , “Impact of MYC in Regulation of Tumor Cell Metabolism,” Biochimica Et Biophysica Acta 1849, no. 5 (2015): 563–569. 25038584 10.1016/j.bbagrm.2014.07.004

[18] A. Wolfer , B. S. Wittner , D. Irimia , et al., “MYC Regulation of a “Poor‐prognosis” Metastatic Cancer Cell state,” Proceedings in the National Academy of Sciences of the United States of America 107, no. 8 (2010): 3698–3703. 10.1073/pnas.0914203107 PMC2840447 20133671

[19] K. J. Savage , N. A. Johnson , S. Ben‐Neriah , et al., “MYC Gene Rearrangements Are Associated With a Poor Prognosis in Diffuse Large B‐cell Lymphoma Patients Treated With R‐CHOP Chemotherapy,” Blood 114, no. 17 (2009): 3533–3537. 19704118 10.1182/blood-2009-05-220095

[20] H. Chen , H. Liu , and G. Qing , “Targeting Oncogenic Myc as a Strategy for Cancer Treatment,” Signal Transduction and Targeted Therapy 3 (2018): 5. 29527331 10.1038/s41392-018-0008-7 PMC5837124

[21] K. I. Chan , S. Zhang , G. Li , et al., “MYC Oncogene: A Druggable Target for Treating Cancers With Natural Products,” Aging and Disease 15, no. 2 (2024): 640–697. 37450923 10.14336/AD.2023.0520 PMC10917530

[22] M. J. Duffy and J. Crown , “Drugging “Undruggable” Genes for Cancer Treatment: Are We Making Progress?,” International Journal of Cancer 148, no. 1 (2021): 8–17. 32638380 10.1002/ijc.33197

[23] D. Lama , T. Vosselman , C. Sahin , et al., “A Druggable Conformational Switch in the c‐MYC Transactivation Domain,” Nature Communications 15, no. 1 (2024): 1865. 10.1038/s41467-024-45826-7 PMC10904854 38424045

[24] L. A. Carabet , P. S. Rennie , and A. Cherkasov , “Therapeutic Inhibition of Myc in Cancer. Structural Bases and Computer‐Aided Drug Discovery Approaches,” International Journal of Molecular Sciences 20, no. 1 (2018): 120. 30597997 10.3390/ijms20010120 PMC6337544

[25] A. D'Avola , K. Kluckova , and A. J. Finch , “Riches JC. Spotlight on New Therapeutic Opportunities for MYC‐Driven Cancers,” OncoTargets and Therapy 16 (2023): 371–383. 37309471 10.2147/OTT.S366627 PMC10257908

[26] B. Amati , “Myc Degradation: Dancing With Ubiquitin Ligases,” PNAS 101, no. 24 (2004): 8843–8844. 15187232 10.1073/pnas.0403046101 PMC428433

[27] F. Ji , J. Ren , and C. Vincke , “Nanobodies: From Serendipitous Discovery of Heavy Chain‐only Antibodies in Camelids to a Wide Range of Useful Applications,” in Single‐Domain Antibodies: Methods and Protocols, ed. G. Hussack and K. A. Henry (2022), 3–17. 10.1007/978-1-0716-2075-5_1 35157266

[28] S. Muyldermans , “Nanobodies: Natural Single‐domain Antibodies,” Annual Review of Biochemistry 82 (2013): 775–797. 10.1146/annurev-biochem-063011-092449 23495938

[29] Q. Su , W. Shi , X. Huang , et al., “Recent Advances of Nanobody Applications in Diagnosis and Detection,” MedComm – Biomaterials and Applications 2, no. 3 (2023): e54.

[30] P. Merikhian , B. Darvishi , N. Jalili , et al., “Recombinant Nanobody Against MUC1 Tandem Repeats Inhibits Growth, Invasion, Metastasis, and Vascularization of Spontaneous Mouse Mammary Tumors,” Molecular Oncology 16, no. 2 (2022): 485–507. 34694686 10.1002/1878-0261.13123 PMC8763658

[31] M. Liu , L. Li , D. Jin , and Y. Liu , “Nanobody—A Versatile Tool for Cancer Diagnosis and Therapeutics,” Wiley Interdisciplinary Reviews: Nanomedicine and Nanobiotechnology 13, no. 4 (2021): e1697. 33470555 10.1002/wnan.1697

[32] Y. Wu , X. Hao , and M. Li , “Application of Nanobody in Cancer Treatment,” Sheng Wu Gong Cheng Xue Bao = Chinese Journal of Biotechnology 33, no. 7 (2017): 1085–1090. 28869728 10.13345/j.cjb.160491

[33] M. Kijanka , B. Dorresteijn , S. Oliveira , and P. M. P. van Bergen en Henegouwen , “Nanobody‐based Cancer Therapy of Solid Tumors,” Nanomedicine (London) 10, no. 1 (2015): 161–174. 10.2217/nnm.14.178 25597775

[34] J. Xu , K. Xu , S. Jung , et al., “Nanobodies From Camelid Mice and Llamas Neutralize SARS‐CoV‐2 Variants,” Nature 595, no. 7866 (2021): 278–282. 34098567 10.1038/s41586-021-03676-z PMC8260353

[35] W. Kang , C. Ding , D. Zheng , et al., “Nanobody Conjugates for Targeted Cancer Therapy and Imaging,” Technology in Cancer Research & Treatment 20 (2021): 15330338211010117. 33929911 10.1177/15330338211010117 PMC8111546

[36] S. Steeland , R. E. Vandenbroucke , and C. Libert , “Nanobodies as Therapeutics: Big Opportunities for Small Antibodies,” Drug Discovery Today 21, no. 7 (2016): 1076–1113. 27080147 10.1016/j.drudis.2016.04.003

[37] D. M. Copolovici , K. Langel , E. Eriste , and Ü. Langel , “Cell‐penetrating Peptides: Design, Synthesis, and Applications,” ACS Nano 8, no. 3 (2014): 1972–1994. 24559246 10.1021/nn4057269

[38] S. L. Y. Teo , J. J. Rennick , D. Yuen , H. Al‐Wassiti , and A. P. R. Johnston , “Pouton CW. Unravelling Cytosolic Delivery of Cell Penetrating Peptides With a Quantitative Endosomal Escape Assay,” Nature Communications 12, no. 1 (2021): 3721. 10.1038/s41467-021-23997-x PMC8211857 34140497

[39] Y. Xue , H. Jiang , T. Li , et al., “Inhibition of Tumor Growth Using a Conjugated Nanobody That Specifically Targets c‐MYC,” Oncogene 44, no. 35 (2025): 3213–3224. 40617995 10.1038/s41388-025-03486-x

[40] A. S. Farrell and R. C. Sears , “MYC Degradation,” Cold Spring Harbor Perspectives in Medicine 4, no. 11 (2014): a014365. 24591536 10.1101/cshperspect.a014365 PMC3935390

[41] J. Jiang , J. Wang , M. Yue , et al., “Direct Phosphorylation and Stabilization of MYC by Aurora B Kinase Promote T‐cell Leukemogenesis,” Cancer Cell 37, no. 2 (2020): 200–215. 32049046 10.1016/j.ccell.2020.01.001 PMC7321798

[42] M. A. Contreras , Y. Serrano‐Rivero , A. González‐Pose , et al., “Design and Construction of a Synthetic Nanobody Library: Testing Its Potential With a Single Selection Round Strategy,” Molecules (Basel, Switzerland) 28, no. 9 (2023): 3708. 37175117 10.3390/molecules28093708 PMC10180287

[43] J. Yan , P. Wang , M. Zhu , et al., “Characterization and Applications of Nanobodies Against human Procalcitonin Selected From a Novel Naïve Nanobody Phage Display Library,” Journal of Nanobiotechnology 13 (2015): 33. 25944262 10.1186/s12951-015-0091-7 PMC4475299

[44] I. Zimmermann , P. Egloff , C. A. J. Hutter , et al., “Generation of Synthetic Nanobodies Against Delicate Proteins,” Nature Protocols 15, no. 5 (2020): 1707–1741. 32269381 10.1038/s41596-020-0304-x PMC7617899

[45] H. Xu , L. Li , B. Deng , et al., “Construction of a T7 Phage Display Nanobody Library for Bio‐panning and Identification of Chicken Dendritic Cell‐specific Binding Nanobodies,” Scientific Reports 12, no. 1 (2022): 12122. 35840654 10.1038/s41598-022-16378-x PMC9284966

[46] E. Pardon , T. Laeremans , S. Triest , et al., “A General Protocol for the Generation of Nanobodies for Structural Biology,” Nature Protocols 9, no. 3 (2014): 674–693. 24577359 10.1038/nprot.2014.039 PMC4297639

[47] C. G. Acevedo‐Rocha , M. T. Reetz , and Y. Nov , “Economical Analysis of Saturation Mutagenesis Experiments,” Scientific Reports 5 (2015): 10654. 26190439 10.1038/srep10654 PMC4507136

[48] S. Tsukiji and T. Nagamune , “Sortase‐mediated Ligation: A Gift From Gram‐positive Bacteria to Protein Engineering,” Chembiochem 10, no. 5 (2009): 787–798. 19199328 10.1002/cbic.200800724

[49] M. W. Popp and H. L. Ploegh , “Making and Breaking Peptide Bonds: Protein Engineering Using Sortase,” Angewandte Chemie (International ed in English) 50, no. 22 (2011): 5024–5032. 21538739 10.1002/anie.201008267

[50] M. A. Gregory and S. R. Hann , “c‐Myc Proteolysis by the Ubiquitin‐proteasome Pathway: Stabilization of c‐Myc in Burkitt's Lymphoma Cells,” Molecular and Cellular Biology 20, no. 7 (2000): 2423–2435. 10713166 10.1128/mcb.20.7.2423-2435.2000 PMC85426

[51] E. Garralda , M. E. Beaulieu , V. Moreno , et al., “MYC Targeting by OMO‐103 in Solid Tumors: A Phase 1 Trial,” Nature Medicine 30, no. 3 (2024): 762–771. 10.1038/s41591-024-02805-1 PMC10957469 38321218

[52] X. Yin , C. Giap , and J. S. Lazo , “Prochownik EV. Low Molecular Weight Inhibitors of Myc‐Max Interaction and Function,” Oncogene 22, no. 40 (2003): 6151–6159. 13679853 10.1038/sj.onc.1206641

[53] H. Han , A. D. Jain , M. I. Truica , et al., “Small‐molecule MYC Inhibitors Suppress Tumor Growth and Enhance Immunotherapy,” Cancer Cell 36, no. 5 (2019): 483–497.e15. 31679823 10.1016/j.ccell.2019.10.001 PMC6939458

[54] J. E. Delmore , G. C. Issa , M. E. Lemieux , et al., “BET Bromodomain Inhibition as a Therapeutic Strategy to Target c‐Myc,” Cell 146, no. 6 (2011): 904–917. 21889194 10.1016/j.cell.2011.08.017 PMC3187920

[55] M. Bekes , D. R. Langley , and C. M. Crews , “PROTAC Targeted Protein Degraders: The Past Is Prologue,” Nature Reviews Drug Discovery 21, no. 3 (2022): 181–200. 35042991 10.1038/s41573-021-00371-6 PMC8765495

[56] L. Kent , Targeting the N‐Myc Oncoprotein Using Nanobody Technology [dissertation] (University of Cambridge, 2018).

[57] M. Welcker , A. Orian , J. Jin , et al., “The Fbw7 Tumor Suppressor Regulates Glycogen Synthase Kinase 3 Phosphorylation‐dependent c‐Myc Protein Degradation,” PNAS 101, no. 24 (2004): 9085–9090. 15150404 10.1073/pnas.0402770101 PMC428477

[58] D. Clift , W. A. McEwan , L. I. Labzin , et al., “A Method for the Acute and Rapid Degradation of Endogenous Proteins,” Cell 171, no. 7 (2017): 1692–1706.e18. 29153837 10.1016/j.cell.2017.10.033 PMC5733393

[59] R. E. Kontermann , “Strategies for Extended Serum Half‐life of Protein Therapeutics,” Current Opinion in Biotechnology 22, no. 6 (2011): 868–876. 21862310 10.1016/j.copbio.2011.06.012

[60] A. Sahni , Z. Qian , and D. Pei , “Cell‐penetrating Peptides Escape the Endosome by Inducing Vesicle Budding and Collapse,” Acs Chemical Biology 15, no. 9 (2020): 2485–2492. 32786250 10.1021/acschembio.0c00478 PMC7512842

