# Supplementary material for: Nanobody‐Mediated c‐MYC Degradation Inhibits Tumor Cell Progression
Source: MedComm (2020). 2026 Mar 26;7(4):e70701. doi: 10.1002/mco2.70701 (PMC13042492; doi:10.1002/mco2.70701)
Supplement: Supplementary file 1 — Supporting Figure 1: Purification and Characterization of Nanobodies.(A‐F) Size‐exclusion chromatography and SDS‐PAGE analysis of purified nanobodies, including M4,M10, M14, M41, Sortase A and MAX. The chromatograms and gels demonstrate the purity and integrity of the nanobodies.(G) Western blot analysis showing the results of a pull‐down assay where c‐MYC was extracted from 293T cell lysate using either purified nanobodies or no nanobodies (negative control). The blot confirms the presence of c‐MYC specifically pulled down by the nanobodies. Supporting Figure 2: Analysis of M4 and CPM4 Effects. (A) Cell viability of HCT116, HepG2, A549, and MDA‐MB‐231 cells treated with CPP, with PBS vehicle as the control. (B) c‐MYC and MAX were incubated with various concentrations of CPM4, followed by addition of a biotin‐labeled E‐box probe for electrophoretic mobility shift assay (EMSA). (C) MALDI–TOF–mass spectra confirming conjugation of M4 with a CPP. Supporting Figure 3: Histopathological assessment of major organs following CPM4 administration. Representative hematoxylin and eososin (H&E)‐stained sections of heart, liver, spleen, lung, kidney, and small intestine collected from mice treated with vehicle (PBS) or CPM4 (treatment regimen as described in Methods). Scale bar, 0.1 mm. Supporting Table 1: Antibodies used in this study. [file MCO2-7-e70701-s001.docx]

**Nanobody-Mediated c-MYC Degradation Inhibits Tumor Cell Progression**

Yuanyuan Xue^1, #^, Hao Jiang^1, #^, Zhaoyun Zong^1^, Xiaolin Tian^1^, Zelong Miao^1^, Ting Li^1^, Yali Wei^2^, Haiteng Deng^1,3*^

1. MOE Key Laboratory of Bioinformatics, Center for Synthetic and Systematic Biology, School of Life Sciences, Tsinghua University, Beijing, China.
2. Department of Clinical Laboratory, Shandong Provincial Hospital, Jinan, China.
3. Zhejiang Key Laboratory of Multiomics and Molecular Enzymology, Yangtze Delta Region Institute of Tsinghua University, Jiaxing, China.

**^*^To whom correspondence should be addressed:**

Haiteng Deng, Prof

Tel: 8610-62790498

E_mail: dht@mail.tsinghua.edu.cn

**^#^These authors contribute equally to this work.**


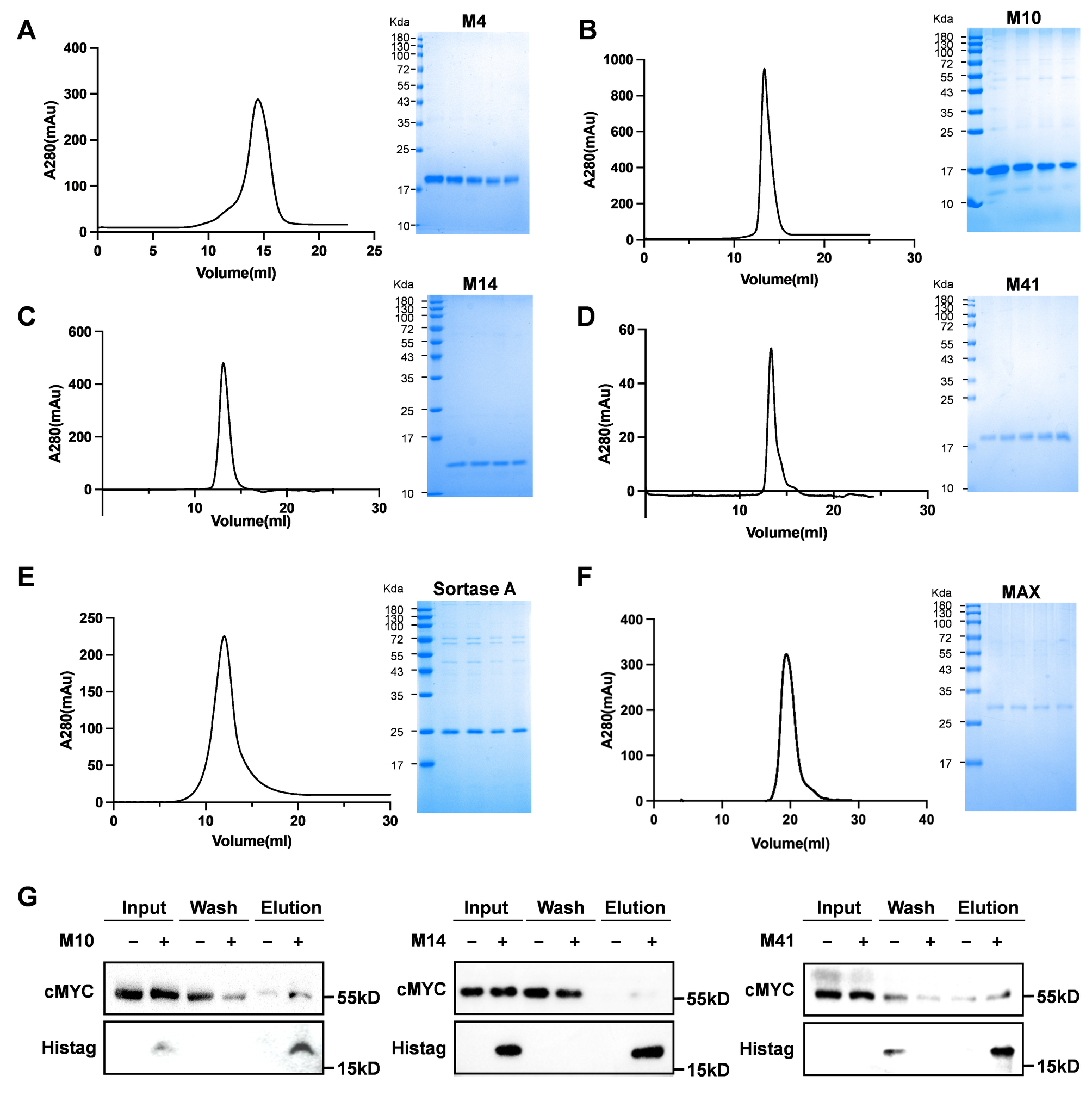


**Figure S1. Purification and Characterization of Nanobodies.**(A-F) Size-exclusion chromatography and SDS-PAGE analysis of purified nanobodies, including M4,M10, M14, M41, Sortase A and MAX. The chromatograms and gels demonstrate the purity and integrity of the nanobodies.(G) Western blot analysis showing the results of a pull-down assay where c-MYC was extracted from 293T cell lysate using either purified nanobodies or no nanobodies (negative control). The blot confirms the presence of c-MYC specifically pulled down by the nanobodies.


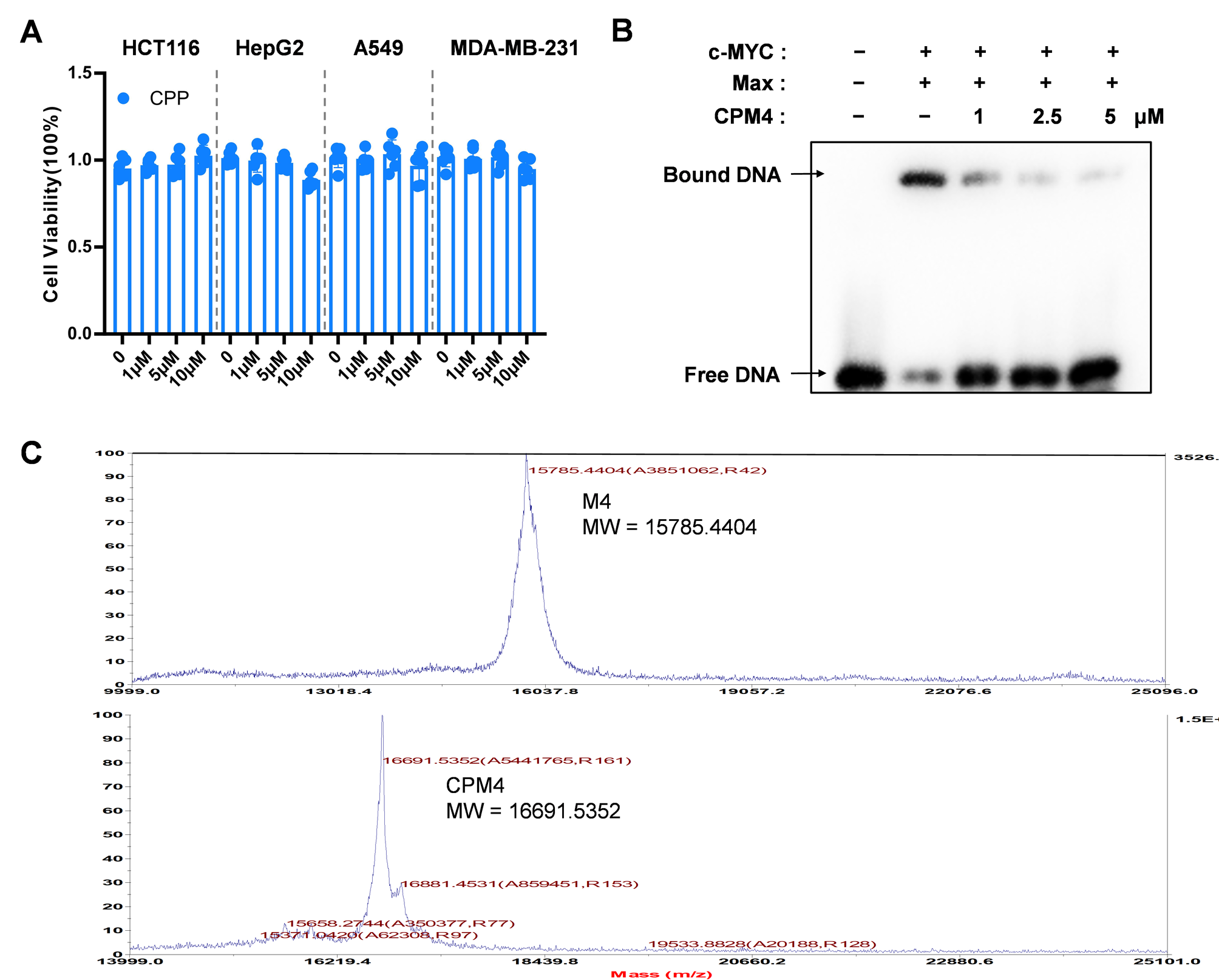


**Figure S2. Analysis of M4 and CPM4 Effects.**(A) Cell viability of HCT116, HepG2, A549, and MDA-MB-231 cells treated with CPP, with PBS vehicle as the control. (B) c-MYC and Max were incubated with various concentrations of CPM4, followed by addition of a biotin-labeled E-box probe for electrophoretic mobility shift assay (EMSA). (C) MALDI-TOF mass spectra confirming conjugation of M4 with a CPP.


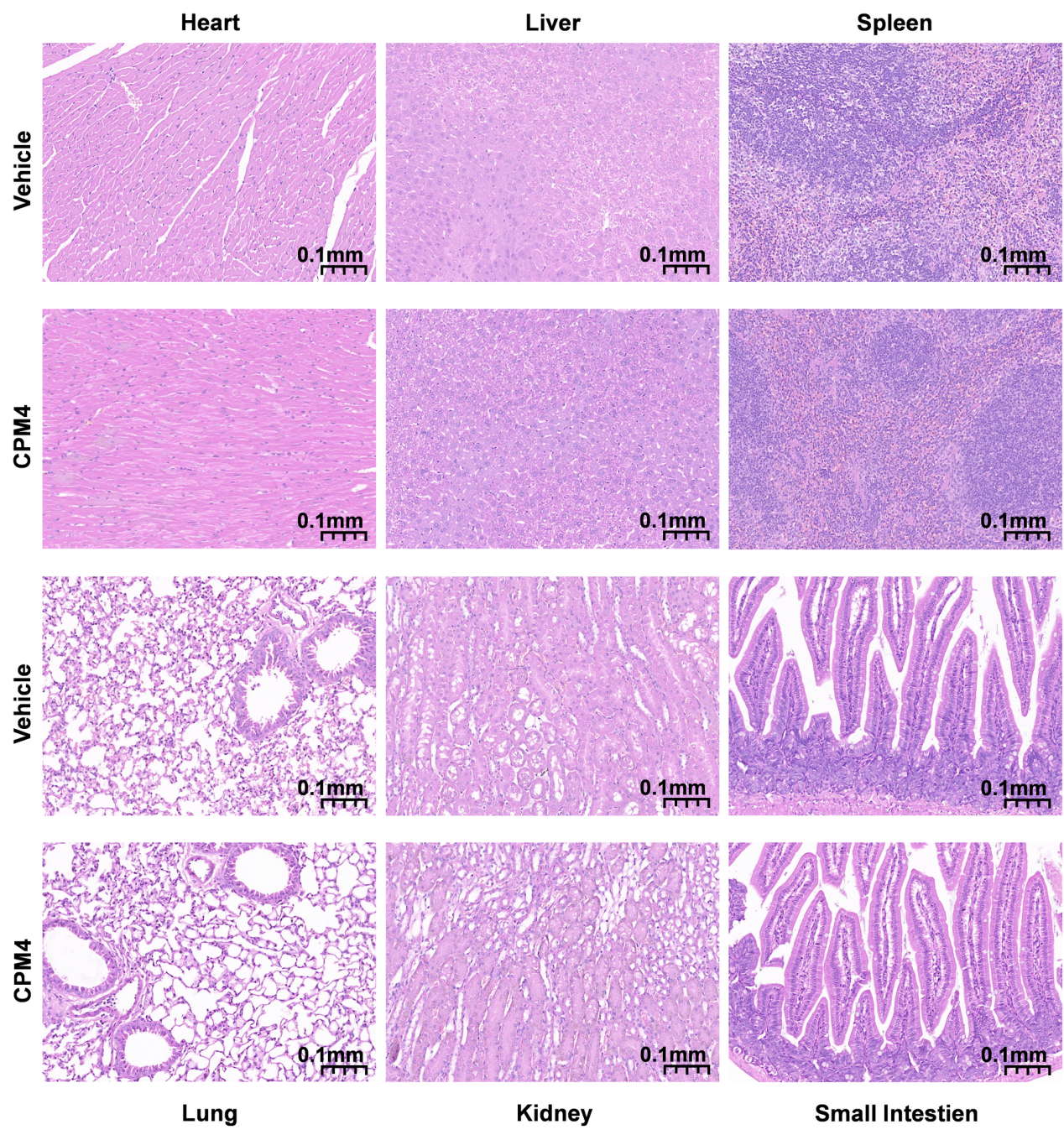


**Figure S3**. **Histopathological assessment of major organs following CPM4 administration.** Representative hematoxylin and eososin (H&E)-stained sections of heart, liver, spleen, lung, kidney, and small intestine collected from mice treated with vehicle (PBS) or CPM4 (treatment regimen as described in Methods). Scale bar, 0.1 mm.

**Supplementary Methods**

**S1. Detailed protocol for c-MYC expression and purification**

The human c-MYC coding sequence was subcloned into the pET28a expression vector to generate a fusion construct with a C-terminal hexahistidine affinity tag. The recombinant plasmid was transformed into *E. coli* for protein production. Bacterial cultures were grown to appropriate density, and cells were harvested by centrifugation. Cell pellets were resuspended in lysis buffer (25 mM Tris-HCl pH 8.0, 250 mM NaCl, 20 mM imidazole, 1 mM dithiothreitol [DTT], 1% Triton X-100) and disrupted by probe-type ultrasonication on ice. The lysate was clarified by centrifugation at 12,000 rpm for 30 min at 4 °C. The pellet fraction containing inclusion bodies was retained, and c-MYC accumulation was verified by 10% SDS-PAGE. For solubilization, inclusion body pellets were washed, weighed, and dissolved in denaturing buffer (25 mM Tris-HCl pH 8.0, 250 mM NaCl, 8 M urea) at approximately 20 mL per gram of wet pellet. The suspension was stirred gently overnight at ambient temperature, then clarified by centrifugation at 17,000 rpm for 30 min at 4 °C. The supernatant containing denatured c-MYC was gradually introduced into ice-cold refolding buffer (25 mM Tris pH 7.5, 150 mM NaCl, 6% glycerol) with continuous stirring to promote protein renaturation. Aggregated material was removed by centrifugation. Refolded protein was captured on Ni-NTA agarose resin pre-equilibrated in lysis buffer (1 h incubation at 4 °C with gentle rotation). After washing with buffer containing 25 mM Tris-HCl pH 8.0, 250 mM NaCl, 20 mM imidazole, and 1 mM DTT, bound protein was eluted with increasing imidazole concentrations (100, 200, 300, and 500 mM). Fractions containing c-MYC were pooled, dialyzed overnight against 1×PBS supplemented with 150 mM NaCl and 2 mM DTT, and further purified by gel filtration on a HiLoad 16/600 Superdex 200 column equilibrated with 1× PBS containing 1 mM DTT. Peak fractions were concentrated using centrifugal ultrafiltration devices (Millipore), snap-frozen in liquid nitrogen, and stored at -80 °C.

**S2. Synthetic nanobody library construction**

The VHH scaffold was derived from camelid heavy-chain-only antibodies. Natural VHH sequences were aligned to define framework regions (FR1-FR4) and CDR boundaries. A consensus framework was designed, codon-optimized for*E. coli* expression, synthesized commercially (Genewiz), and cloned into pCantab 5E. Library diversity was introduced through sequential PCR amplifications: (i) FR1-CDR1-FR2 fragment with 5′ SfiI site and NNK codons in CDR1; (ii) FR2-CDR2-FR3-CDR3-FR4 fragment with NNK codons in CDR2 and CDR3; (iii) overlap-extension assembly of full-length VHH with 3′ NotI site.The final product was gel-purified, digested with SfiI and NotI, ligated into pCantab 5E, and electroporated into*E. coli* TG1.Library capacity was estimated by serial dilution plating (10⁻¹ to 10⁻⁷) on selective 2×TY agar. Sequence diversity was assessed by colony PCR and sequencing of 100 randomly selected clones.

**S3. Phage display panning protocol**

Immunotubes were coated overnight at 4 °C with 4 mL of c-MYC protein (50 μg/mL) or BSA (negative control). After washing three times with PBST (PBS + 0.05% Tween-20), tubes were blocked with 4 mL of 2% (w/v) non-fat milk in PBS for 2 h at 37 °C.Amplified phage library (~5×10¹² CFU) diluted in 4 mL PBS containing 2% milk was added to c-MYC-coated tubes and incubated for 2 h at 37 °C. Unbound phages were removed by sequential washing (5× PBST, 5× PBS). Bound phages were eluted with 1 mL of 0.1 M triethylamine (10 min with gentle agitation), immediately neutralized with 0.5 mL of 1 M Tris-HCl pH 7.4, and transferred to ice. Eluted phages were used to infect 16 mL of exponentially growing *E. coli* TG1 (OD₆₀₀ ≈ 0.5). Additionally, 4 mL of TG1 culture was added directly to immunotubes and incubated at 37 °C for 45 min with gentle shaking; cultures were then combined. Phage titers were determined by serial dilution plating (10⁻¹ to 10⁻⁷) on 2×TYE/ampicillin agar. The remaining culture was concentrated, spread onto 2×TYE plates (100 μg/mL ampicillin, 0.1% glucose), and incubated overnight at 37 °C. Bacterial lawns were harvested and used to inoculate 250 mL of 2×TY medium (ampicillin + glucose), cultured to OD₆₀₀ ≈ 0.6.M13KO7 helper phage was added at a 1:20 multiplicity of infection and incubated at 37 °C for 45 min. Cells were pelleted (8,500 rpm, 15 min, 4 °C), resuspended in 2×TY containing 100 μg/mL ampicillin and 70 μg/mL kanamycin, and cultured overnight at 30 °C.Phages were precipitated from clarified supernatant by adding one-quarter volume of PEG/NaCl solution (20% PEG 8000, 2.5 M NaCl) and incubating at 4 °C for 2 h. The pellet was resuspended in PBS, clarified by centrifugation (12,000 rpm, 20 min), aliquoted with 15% glycerol, and stored at −80 °C. Four panning rounds were performed with progressively stringent washing.

**S4. Phage ELISA screening**

Sixty colonies from the final panning round were cultured in 5 mL of 2×TY/ampicillin at 37 °C (220 rpm) to OD₆₀₀ ≈ 0.6. One-milliliter aliquots were transferred to tubes, superinfected with M13KO7 (1:20 ratio), and incubated at 37 °C for 45 min. Cells were pelleted (10,000 rpm, 10 min, 4 °C), resuspended in 1 mL of 2×TY containing ampicillin and kanamycin, and cultured overnight at 30 °C (180 rpm). Phage-containing supernatants were harvested for ELISA.For ELISA, 96-well plates were coated with c-MYC (50 μg/mL) or BSA overnight at 4 °C, washed (3× PBST, 3× PBS), and blocked with 200 μL of 2% milk for 2 h at 37 °C. After washing, 100 μL of phage supernatant was added per well (2 h, 37 °C). Bound phages were detected with HRP-conjugated anti-M13 antibodies (1 h, room temperature). Following extensive washing, 100 μL TMB substrate was added (5 min), and the reaction was stopped with 50 μL of 1 M H₂SO₄. Absorbance at 450 nm was measured. Clones with signal ≥2-fold above BSA background were scored as positive.

**S5. Nanobody expression and purification**

Nanobody sequences were cloned into pET-21b and transformed into *E.coli* BL21(DE3). Single colonies were inoculated into LB/ampicillin medium and grown overnight at 37 °C. Expression was induced with IPTG during exponential growth. Cells were harvested by centrifugation and lysed in buffer (25 mM Tris-HCl pH 8.0, 250 mM NaCl, 1 mM PMSF) by probe sonication. Clarified lysate was applied to Ni-NTA agarose resin (GE Healthcare). After washing, bound nanobodies were eluted with stepwise imidazole gradients. Fractions were analyzed by SDS-PAGE. Nanobody-containing fractions were polished by size-exclusion chromatography on a Superdex 75 Increase 10/300 GL column (Cytiva) using an ÄKTA system. Peak fractions were pooled, concentrated with centrifugal filters, quantified spectrophotometrically, aliquoted, snap-frozen, and stored at -80 °C.

**S6. Sortase A-mediated conjugation**

GGG-CPP peptide (Bankpeptide Biological Technology) was dissolved in 50 mM Tris buffer pH 8.0. Conjugation reactions contained nanobody-LPETG-His₆ (20 μM), GGG-CPP (50 μM), and Sortase A (50 μM) in reaction buffer (50 mM Tris-HCl pH 7.5, 150 mM NaCl, 10 mM CaCl₂). Reactions proceeded at 30 °C for 16 h. Unreacted His-tagged components were removed by Ni-NTA capture. Excess GGG-CPP was eliminated by repeated ultrafiltration using 10 kDa MWCO devices (Millipore) with six buffer exchanges (20 mM Tris-HCl pH 7.5, 500 mM NaCl, 20% glycerol). Conjugate mass and purity were verified by MALDI-TOF-MS under reducing conditions.

**S7. Surface plasmon resonance parameters**

SPR experiments were performed on a Biacore 3000 instrument. c-MYC was immobilized on CM5 sensor chips via amine coupling: surface activation with 100 mM NHS and 400 mM EDC; c-MYC injection (4 μg/mL in 10 mM sodium acetate pH 4.5) to achieve ~1,400 ± 53 RU; capping with 1 M ethanolamine-NaOH pH 8.5. Nanobodies were prepared as twofold serial dilutions (1-50 nM) in PBS containing 0.005% Tween-20. Measurements were conducted at 25 °C with 20 μL/min flow rate. Association and dissociation phases were each monitored for 180 s. Surface regeneration was performed with two injections of 20 mM glycine-HCl pH 2.0. Sensorgrams were globally fitted to a 1:1 Langmuir model using BIAevaluation software.

**S8. Confocal microscopy staining protocol**

HCT116 cells were treated with 10 μM M4 or CPM4 for 24 h at 37 °C. Cells were washed with PBS, seeded onto poly-L-lysine-coated confocal dishes (Fisher Scientific, #80824), and allowed to adhere for 1 h at 37 °C. Fixation was performed with 4% paraformaldehyde (10 min, room temperature), followed by permeabilization with PBST (PBS + 0.1% Triton X-100, 5 min). Cells were incubated overnight at 4 °C with primary antibodies: anti-HA tag (Cell Signaling, #3724) and anti-c-MYC (Cell Signaling, #9367). After three PBST washes, cells were incubated for 1 h at room temperature with secondary antibodies: Alexa Fluor 488-conjugated goat anti-rabbit IgG (Thermo Scientific, #A32371) and Alexa Fluor 647-conjugated goat anti-mouse IgG (Thermo Scientific, #A21235). Nuclei were counterstained with DAPI (1 μg/mL, 10 min).Images were acquired on a Nikon A1R HD25 confocal microscope using a 100× oil-immersion objective with 488 nm and 653 nm laser excitation.

**S9. HDX-MS detailed protocol**

For deuterium labeling, 5 μL of protein solution (180 μM) was mixed with 45 μL of labeling buffer (20 mM Tris, 500 mM (NH₄)₂SO₄, 99% D₂O, pH 8.5) and incubated at 25 °C. Reactions were quenched by adding 50 μL of ice-cold quench buffer (4 M guanidine-HCl, 200 mM citric acid, 500 mM TCEP, pH 1.8) and immediate placement on ice. Samples were digested with 5 μL of 1 μM pepsin (2 min) and injected into a Thermo-Dionex Ultimate 3000 HPLC system.Peptides were separated on an Acquity UPLC BEH C18 column (1.7 μm, 2.1 × 50 mm; Waters) using a 20-min linear gradient at 115 μL/min (mobile phase A: water + 1% formic acid; mobile phase B: acetonitrile + 1% formic acid). Eluting peptides were analyzed on a Q Exactive mass spectrometer in data-dependent acquisition mode. Survey Scans (m/z 350-2000) were acquired at 70,000 resolution with ion source at 25 °C and spray voltage of 3.0 kV.Peptide identification was performed using Proteome Discoverer (v1.4) with no-enzyme search, allowing up to two missed cleavages. Deuterium uptake was quantified using HDExaminer software.

**S10. Cell viability and apoptosis detailed protocols**

CCK-8 assay: Cells (1 × 10³ per well) were seeded in 96-well plates and treated with indicated M4 or CPM4 concentrations. At endpoint, 10 μL CCK-8 reagent (KeyGEN BioTECH) was added per well and incubated for 2 h at 37 °C. Absorbance at 450 nm was measured using a microplate reader.Apoptosis detection: Treated cells were harvested, washed with PBS, and stained using an Annexin V-APC/7-AAD apoptosis detection kit (KeyGEN BioTECH) according to the manufacturer's protocol. Samples were analyzed by flow cytometry (BD Biosciences) to determine early apoptotic (Annexin V⁺/7-AAD⁻) and late apoptotic (Annexin V⁺/7-AAD⁺) populations.

**S11. Immunohistochemistry protocol**

Formalin-fixed tumors were paraffin-embedded and sectioned at 4 μm thickness. Sections were deparaffinized in xylene, rehydrated through graded ethanol, and subjected to antigen retrieval in citrate buffer (pH 6.0) at 95 °C for 20 min. Endogenous peroxidase was blocked with 3% H₂O₂. Sections were blocked with 5% BSA (30 min) and incubated overnight at 4 °C with primary antibodies: anti-c-MYC (1:200; Cell Signaling, #9367), anti-PCNA (1:500; Abcam, #ab29), or anti-Ki-67 (1:200; Cell Signaling, #9129). Following washing, sections were incubated with HRP-conjugated secondary antibodies (1 h, room temperature). Immunoreactivity was visualized with DAB chromogen, and sections were counterstained with Mayer's hematoxylin.

**Table S1. Antibodies used in this study**

| Target | Catalog Number | Supplier | Application | Dilution |
| --- | --- | --- | --- | --- |
| c-MYC | 9367 | Cell Signaling Technology | WB, IHC, IF | 1:1000 (WB), 1:200 (IHC/IF) |
| MAX | ab227298 | Abcam | WB | 1:1000 |
| His-HRP | 105327-MM02T-H | SinoBiological | WB | 1:5000 |
| Phospho-c-MYC  (Thr58) | ab185655 | Abcam | WB | 1:1000 |
| Phospho-c-MYC (Ser62) | ab185656 | Abcam | WB | 1:1000 |
| Cleaved Caspase-3 (Asp175) | 9661S | Cell Signaling Technology | WB | 1:1000 |
| Cleaved Caspase-9 | HY-P80964 | MedChemExpress | WB | 1:1000 |
| GSK-3β | 9315 | Cell Signaling Technology | WB | 1:1000 |
| Bcl-2 | HY-P80566 | MedChemExpress | WB | 1:1000 |
| Bax | HY-P80028 | MedChemExpress | WB | 1:1000 |
| Histone H3 | 9715 | Cell Signaling Technology | WB | 1:2000 |
| β-actin | 9100026001 | ABclonal | WB | 1:5000 |
| HA-tag | 3724 | Cell Signaling Technology | IF | 1:200 |
| PCNA | ab29 | Abcam | IHC | 1:500 |
| Ki-67 | 9129 | Cell Signaling Technology | IHC | 1:200 |
| Anti-rabbit IgG-HRP | 7074S | Cell Signaling Technology | WB | 1:3000 |
| Anti-mouse IgG-HRP | 7076S | Cell Signaling Technology | WB | 1:3000 |
| Alexa Fluor 488 anti-rabbit | A32371 | Thermo Fisher Scientific | IF | 1:500 |
| Alexa Fluor 647 anti-mouse | A21235 | Thermo Fisher Scientific | IF | 1:500 |
| HRP-anti-M13 | - | - | Phage ELISA | 1:5000 |
